# LMO2 promotes the development of AML through interaction with transcription co-regulator LDB1

**DOI:** 10.1038/s41419-023-06039-w

**Published:** 2023-08-12

**Authors:** Lihui Lu, Jianwei Wang, Fang Fang, Ailian Guo, Shuting Jiang, Yanfang Tao, Yongping Zhang, Yan Li, Kunlong Zhang, Zimu Zhang, Ran Zhuo, Xinran Chu, Xiaolu Li, Yuanyuan Tian, Li Ma, Xu Sang, Yanling Chen, Juanjuan Yu, Yang Yang, Haibo Cao, Jizhao Gao, Jun Lu, Shaoyan Hu, Jian Pan, Hailong He

**Affiliations:** 1 Children’s Hospital of Soochow University, Suzhou, 215003 China; 2 Department of Pediatrics, The Affiliated Hospital of Xuzhou Medical University, Xuzhou, 221000 China; 3 Institute of Pediatric Research, Children’s Hospital of Soochow University, Suzhou, 215003 China; 4 Department of Hematology, Children’s Hospital of Soochow University, Suzhou, 215003 China; 5 CAS Key Laboratory of Genomic and Precision Medicine, Beijing Institute of Genomics, Chinese Academy of Sciences and China National Center for Bioinformation, Beijing, 100101 China; 6 Department of Pediatric Surgery, The Affiliated Hospital of Yangzhou University, Yangzhou, 225000 China

**Keywords:** Paediatric cancer, Oncogenes

## Abstract

One of the characteristics of leukemia is that it contains multiple rearrangements of signal transduction genes and overexpression of non-mutant genes, such as transcription factors. As an important regulator of hematopoietic stem cell development and erythropoiesis, LMO2 is considered an effective carcinogenic driver in T cell lines and a marker of poor prognosis in patients with AML with normal karyotype. LDB1 is a key factor in the transformation of thymocytes into T-ALL induced by LMO2, and enhances the stability of carcinogenic related proteins in leukemia. However, the function and mechanism of LMO2 and LDB1 in AML remains unclear. Herein, the LMO2 gene was knocked down to observe its effects on proliferation, survival, and colony formation of NB4, Kasumi-1 and K562 cell lines. Using mass spectrometry and IP experiments, our results showed the presence of LMO2/LDB1 protein complex in AML cell lines, which is consistent with previous studies. Furthermore, in vitro and in vivo experiments revealed that LDB1 is essential for the proliferation and survival of AML cell lines. Analysis of RNA-seq and ChIP-Seq results showed that LDB1 could regulate apoptosis-related genes, including LMO2. In LDB1-deficient AML cell lines, the overexpression of LMO2 partially compensates for the proliferation inhibition. In summary, our findings revealed that LDB1 played an important role in AML as an oncogene, and emphasize the potential importance of the LMO2/LDB1 complex in clinical treatment of patients with AML.

## Introduction

Acute myeloid leukemia (AML) is a hematological tumor disease with genetic heterogeneity caused by carcinogenic transformation of hematopoietic progenitor cells in the bone marrow [1, 2]. Multiple gene mutations, increased expression of transcription factors, and chromosome rearrangement have been detected in the primitive cells of patients with AML [3]. For example, mutations in transcription factors RUNX1 and C/EBPA may affect the development of hematopoietic stem cells and are essential for myeloid terminal differentiation [4]. The transcription factor complex of AML1-ETO fusion protein, consisting of AML1-ETO, LMO2, LDB1 and LYL1, has recently been considered the key to leukemia maintenance and differentiation blocking, because knockout of this complex can delay leukemogenesis in mice [5]. Thus, it is inferred that transcription factors play important role in the occurrence and development of leukemia, and identification of novel molecular targets is a promising strategy for the clinical treatment of leukemia patients.

LMO2 (LIM-onlyprotein2) is considered to be a key transcriptional regulator in hematopoietic stem cell development and erythropoiesis [6]. The LIM domain of the LMO2 protein can mediate protein-protein interaction, while curl, zinc finger and SH3 are the favorable interaction domains of LMO2. In addition, members containing these domains have significantly abundant functions in cancer, speculating that the main functions of LMO2 are involved in carcinogenesis [7]. Previous studies have shown that LMO2 mutations in the non-coding sites of T-ALL cell lines could result in the formation of abnormal promoters and enhancers, which drive the overexpression of LMO2 in patients with T-ALL [8]. Thymic progenitor cells overexpressing LMO2 have stem cell characteristics, clonal expansion and subsequent oncogenic-driven mutations, which eventually lead to leukemia [9]. It has been reported that the high expression of LMO2 in normal karyotype patients with AML is associated with poor survival rates [10]. However, the functions and molecular mechanisms of LMO2 in AML cell lines have not been thoroughly investigated.

LDB1 is a LIM domain-binding protein that interacts with LMO2/GATA1 and SCL/TAL1 in erythroid cells to form the LDB1 complex [11]. The protein complex has multiple functions in the regulation of gene expression, among which the DNA loop structure of LDB1 plays an important role in the regulation of erythroid genes by mediating the communication between remote enhancer and promoter. An additional feature of this complex is the formation of a core complex with SSbp to assemble enhancers to integrate transcription factors [12]. The T-ALL LMO2 transgenic mouse model showed that the Ldb1/Lmo2 transcriptional complex could promote the self-renewal of hematopoietic progenitor cells. LDB1 is a key factor in thymocyte radiation resistance and tumor transformation into T-ALL induced by LMO2, which determines the key role of non-proto-oncogenes in leukemia [13]. The role of LDB1 complex in erythroid differentiation has been widely studied [14], however, the functions and the underlying mechanisms of LDB1 and LDB1 complex in AML remain unclear.

In our study, we found that LMO2 and LDB1 could promote the proliferation, survival, and colony formation of AML cell lines. Knockdown of the LDB1 gene affected proliferation and apoptosis of AML cell lines and also regulated expression of multiple genes, including LMO2. The reintroduction of LMO2 partially saved the phenotype of proliferation deficiency caused by LDB1 gene knockdown. Our studies confirmed that LDB1 and LMO2 could function as oncogenes in AML cell lines through a mutually regulated manner. LMO2 and LDB1 are likely to be novel regulatory factors in the clinical treatment of patients with AML.

## Materials and methods

### Cell lines and mice

The NB4, Kasumi-1, and K562 human leukemia cell lines were obtained from the cell bank of the Chinese Academy of Science and maintained in complete RPMI 1640 (22400089, Gibco, USA). HEK293T and 293FT cells were obtained from the cell bank of the Chinese Academy of Science and maintained in complete DMEM medium (12430054, Gibco, USA). BALB/c mice were purchased from Cavens Model Animal Research Inc. All animal studies were approved and licensed by the Animal Care and Use Committee at the Children’s hospital of Soochow University (CAM-SU-AP#:JP-2018-1). All cell lines were regularly authenticated by STR profiling and tested to confirm the absence of mycoplasma infection.

### Samples

Clinical specimens were collected from bone marrow samples of acute leukemia patients in the affiliated Children’s Hospital of Suzhou University, including 22 cases of acute myeloid leukemia, B-ALL 4 cases and T-ALL 4 cases. Prior to the study, written informed consent was obtained from the guardian of each participant. The ethics committee of the Children’s Hospital of Suzhou University approved the study (numbered SUEC2000-021 and SUEC2011-037). Primary AML samples were obtained from the bone marrow of pediatric AML patients and acute myeloid leukemia cells were isolated by density gradient centrifugation. The patient information is shown in Supplementary Table 1.

### Lentivirus preparation and infection

The lentiviral vector Plko.1 (IGE Biotechnology LTD, China) was used for knockdown. Gene overexpression was achieved by inserting the CDS region of the LDB1 or LMO2 transcript into the pLVX-EF1α-Puro plasmid. After sequencing, purified plasmids were transfected into 293FT cells together with packaging plasmids, psPAX2 and pMD2G, using PEI(49553-93-7, Sigma-Aldrich, USA) reagent with a ratio of 4:3:1 according to the manufacturer’s protocol. The supernatants were collected after 48 h for virus purification. Cells were then infected with purified virus for 24 h in complete 1640 culture medium. Consequently, the infected cells were cultured with 2 ug/ml puromycin (ST551, Beyotime, China) for 48 h and used for cell proliferation, apoptosis, cell cycle and other experiments. Murine P388D1 cells with LDB1 interference were first transfected with luciferase and then screened with G418 at a work concentration of 400 ug/ml (108321-42-2, Selleck, USA). 2*10^5^ P388D1 cells were injected into the tail vein of each mouse.

The sequences of shRNA were as follows:

LDB1-shRNA-1: 5′-CCGGGGACAGAGGAGTGTGACAATCCTCGAGGATTGTCACACTCCTCTGTCCTTTTTGAATT-3′

LDB1-shRNA-2: 5′-CCGGGCTACTTCCGCAGCATCTTTGCTCGAGCAAAGATGCTGCGGAAGTAGCTTTTTGAATT-3′

LDB1-shRNA-3: 5′-CCGGGGATAAAGACGTGGCACTTCACTCGAGTGAAGTGCCACGTCTTTATCCTTTTTGAATT-3′

LMO2-shRNA-1: 5′-CCGGGCTCTGCCGGAGAGACTATCTCTCGAGAGATAGTCTCTCCGGCAGAGCTTTTTGAATT-3′

LMO2-shRNA-2: 5′-CCGGGGATTCGTGCCTATGAGATGACTCGAGTCATCTCATAGGCACGAATCCTTTTTGAATT-3′

LMO2-shRNA-3: 5′-CCGGGGACTAAGATCAATGGGATGACTCGAGTCATCCCATTGATCTTAGTCCTTTTTGAA-3′

Mouse-Ldb1-shRNA-1: 5′-CCGGGCACGGCAAACCCATGTTTACCTCGAGGTAAACATGGGTTTGCCGTGCTTTTTGAA-3′

Mouse-Ldb1-shRNA-2: 5′-CCGGGCTGGAGAACACCCAGTTTGACTCGAGTCAAACTGGGTGTTCTCCAGCTTTTTGAA-3′

Mouse-Ldb1-shRNA-3: 5′-CCGGGGACAGAGGAGTGTGACAATCCTCGAGGATTGTCACACTCCTCTGTCCTTTTTGAA-3′

### Real-time quantitative PCR

Total RNA was extracted from cells with TRIZOL reagent (15596018, Invitrogen, USA) according to the manufacturer’s protocol. Next, RNA was reverse transcribed using the High-Capacity cDNA Reverse Transcription Kit (4368813, Applied Biosystems, USA) according to the manufacturer’s protocol. Real-time quantitative PCR was performed with a gradient PCR machine (ABI, USA) using FastStartTM Universal SYBR® Green Master Mix (4913850001, Roche, Germany).

The qPCR primers were as follows:

LMO2 forward, 5′-TCTGCCGGAGAGACTATCTCA-3′,

LMO2 reverse, 5′-ATAGGCACGAATCCGCTTGTC-3′;

LDB1 forward, 5′-CAAACGGCTTCAGAACTGGAC-3′,

LDB1 reverse, 5′-TCCGGCCAATGGTATATCTCTT-3′;

GAPDH forward, 5′-TGCACCACCAACTGCTTAG-3′,

GAPDH reverse, 5′-GATGCAGGGATGATGTTC-3′;

Mouse-Ldb1 forward, 5′-AAGTCATTCAAGCTGTACTCGC-3′,

Mouse-Ldb1 reverse, 5′-TCCAGTTCTGTAGCCGTTTGT-3′;

β-Actin forward, 5′-AGCCTTAGCCTGGACCCATA-3′,

β-Actin reverse, 5′-CGGACTCATCGTACTCCTGC-3′;

HHEX forward, 5′-GGCAAACCTCTACTCTGGAGC-3′,

HHEX reverse, 5′-GTCGTTGGAGAATCTCACCTG-3′;

NFE2 forward, 5′-CCACCACCCACAACTTACTG-3′,

NFE2 reverse, 5′-GAGGGCTAAGGGGTCTTGGA-3′;

GATA1 forward, 5′-TGCGGCCTCTATCACAAGATG-3′,

GATA1 reverse, 5′-CTGCCCGTTTACTGACAATCA-3′;

STAT5B forward, 5′-CAGAACACGTATGACCGCTG-3′,

STAT5B reverse, 5′-CTGGAGAGCTACCATTGTTGG-3′;

NFIB forward, 5′-GCTGTGTCTTATCCAATCCCG-3′,

NFIB reverse, 5′-TGCCTTTGAACAGGATCACCA-3′;

TAL1 forward, 5′-TTCCCTATGTTCACCACCAA-3′,

TAL1 reverse, 5′-AAGATACGCCGCACAACTTT-3′;

ZEB2 forward, 5′-GCGATGGTCATGCAGTCAG-3′,

ZEB2 reverse, 5′-CAGGTGGCAGGTCATTTTCTT-3′;

ZFP36L2 forward, 5′-CAACTCCACGCGCTACAAGA-3′,

ZFP36L2 reverse, 5′-CACTTTTCGCCGTACTTGCAC-3′.

### Western blot

The collected cells were rinsed with PBS and lysed by RIPA buffer (Byeotime, China). Equal amounts of protein were separated by SDS-PAGE, transferred to PVDF membranes (CST, USA), blocked with milk, added with primary antibody and shaken at 4 °C overnight. The PVDF membrane was incubated with the secondary antibody at room temperature, and the ECL luminescent solution (Millipore, USA) was added to the membrane for visualization with AI600 image gel imaging analyzer (GE, USA). The following primary antibodies were used: LMO2 (ab91652, abcam, USA), LDB1 (ab96799, abcam, USA), HA (ab9110, abcam, USA), c-Myc (9402, CST, USA), PARP (9542, CST, USA), Flag (14793, CST, USA), GAPDH (5174, CST, USA) as a reference protein.

### Cell proliferation and colony assays

The transfected cells were seeded into 96-well plates at a density of 5*10^3^ cells/well for culture. On the 2nd/4th/6th day after puromycin selection, CCK8 (C0037, Beyotime, China) was added, and the cells were tested by enzyme labeling. A spectrometer (Thermo, USA) measured absorbance at 450 nm. The cells of the experimental and the control groups were inoculated in soft agar medium, and after culturing for 9 to 14 days, the cells were stained with Giemsa (C0131, Beyotime, China), and the number of colonies was counted.

### Apoptosis and cell-cycle assays

Following the manufacturer’s protocol, the samples were washed with PBS, resuspended in 1 × binding buffer, stained with fluorescein isothiocyanate FITC-Annexin V antibody and PI solution using a FITC-Annexin V apoptosis kit (556420, BD, USA), then incubated in the dark for 15 min. Cell apoptosis was analyzed using the Beckman Gallios™ flow cytometer. Samples used for cell cycle assays were fixed overnight with 75% alcohol before addition of RNase A (ST578, Beyotime, China) and PI. After incubation in the dark for 30 min, the cells were assayed by flow cytometry.

### Co-IP

293 T cells transfected with overexpressing LMO2-HA were lysed in a lysate containing NP-40 (P0013F, Beyotime, China) and Protein inhibitor. After centrifugating, the supernatant was collected, a part of it was added to 5× protein loading buffer, and then the protein was denatured by heating at 100 °C and retained as input. The other two parts of the supernatant were mixed with protein A/G beads (B23202, Bimake, China) and HA antibody (ab1424, Abcam, USA) or IgG Antibodies (3420, CST, USA) respectively, and rotated overnight at 4 °C. Then, samples were washed three times, lysed in lysis buffer, resuspended in 1× protein loading buffer, denatured by heat, and the supernatant was taken for WB detection or mass spectrometry analysis. Specific immunoprecipitation was performed in NB4 cells with LDB1 antibody and the proteins in the complexes were detected by WB.

### RNA-seq analysis and data processing

RNA-seq was performed using protocols from Novogene Bioinformatics Technology Co., Ltd. (Beijing, China). Library construction was the first step, with reverse transcription of total RNA to cDNA. Next, the cDNA library was sequenced. We then filtered the raw reads and mapped the clean reads using HISAT. Next we calculated gene expression level and identified differentially expressed genes with DESeq2 (P < 0.05 and fold-change > 2 or fold-change < 0.5). For enrichment analysis, differentially expressed genes were analyzed using the GSEA software (UC San Diego and Broad Institute).

### Chromatin immunoprecipitation (ChIP)

We crosslinked 3–5× 10^7^ cells with 1% formaldehyde for 10 minutes, and then quenched the crosslinking reaction with 1.25 M glycine at room temperature for 5 minutes. Fixed cells were then harvested, lysed, and sonicated with the Bioruptor (Diagenode, Liège, Belgium). Sonicated chromatin was next incubated with an anti-LDB1 antibody (No. ab96799; Abcam, Cambridge, UK) overnight at 4 °C. DNA was eluted and purified with a QIAquick PCR purification kit (cat. No. 208106; Qiagen, Hilden, Germany). Samples were sequenced using the BGISEQ 2000 platform (Beijing Genomics Institute (Shenzhen, China)) and the NovaSeq 6000 platform (Novogene Bioinformatics Technology Co., Ltd. Beijing, China). We aligned the raw ChIP-Seq data to UCSC hg38 (the reference genome) with Bowtie2 (v 2.4.1) [15], according to the alignment parameters -p 4 -q -x. Peaks were then identified with MACS2 (v2.0.10) [16], according to the parameters -g hs -n test -B -q 0.01. Next we converted the bedgraph files generated by MACS2 to bigwig files with the UCSC bedGraphToBigWig tool, and then visualized the bigwig files using Integrative Genomics Viewer (IGV) [17] and WashU tool (http://epigenomegateway.wustl.edu/browser/).

### CUT&Tag

The CUT&Tag Assay was performed on human NB4 cells using the Hyperactive Universal CUT&Tag Assay Kit for Illumina (TD903-01, Vazyme) according to the manufacturer’s instructions [18]. Samples from 2 * 10^6^ cells incubation, join ConA Beads activation, and a fight after blending let stand up for the night. The second antibody diluted at 1:100 was added overnight. After incubation at room temperature, Pa/g-Tnp was added for incubation. Next, the sample was fragmented and DNA extracted for sequencing. The following primary antibodies were used: LDB1 (ab96799, abcam, USA), IgG (ab172730, abcam, USA). Samples were sequenced using the NovaSeq 6000 platform (Novogene Bioinformatics Technology Co., Ltd. Beijing, China). We aligned the raw CUT&Tag data to UCSC hg38 (the reference genome) with Bowtie2 (v 2.4.4) [15], according to the alignment parameters -p 4 -q -x. Peaks were then identified with MACS3 (v3.0) [16], according to the parameters –f BAMPE -g hs -B -q 0.01 –SPMR.

### Public ChIP-Seq data collection and analysis

In the present study, the Cistrome database (http://www.cistrome.org/) was used to search public ChIP-Seq datasets of transcription factors (RUNX1, ERG, FLI1, LMO2, and CEBPA) for AML cell lines. Raw data from these public ChIP-Seq datasets were downloaded and aligned to UCSC hg38 (the reference genome) with Bowtie2 (v 2.3.5) [15], according to the parameters -p 4 -q -x. Peaks were obtained with MACS2 (v2.0.9) [16], according to the parameters -g hs -n test -B -q 0.01.The bigwig files of these datasets (GSE45738, GSE123872, GSE23730, GSE60130, GSE81992, GSE76464, GSE79899) were next visualized using Integrative Genomics Viewer (IGV) [19] and WashU tool (http://epigenomegateway.wustl.edu/browser/).

### Public Hi-C data collection and analysis

Hi-C data for THP-1 cell line (GSE126979) were downloaded from the Gene Expression Omnibus database. Read mapping and loop calling were performed with HiC-pro (v.3.1.0) [19]. For alignment, MboI restriction sites in the hg38 build were used. HiC-pro uses Bowtie2 for mapping and we specified –very-sensitive -L 30 –score-min L, −0.6, −0.2 –end-to-end –reorder for global options and –very-sensitive -L 20 –score-min L, −0.6, −0.2 –end-to-end –reorder for local options. We used ‘GATCGATC’ as the ligation site during the mapping process. The results of this analysis were visualized and graphed using WashU tool (http://epigenomegateway.wustl.edu/browser/).

### Single-cell RNA-seq data analysis

Processed scRNA-seq data of bone marrow aspirates of thirteen AML patients were obtained from Genome Sequence Archive for Human at the BIG data center, Beijing Institute of Genomics, Chinese Academy of Sciences and China National Center for Bioinformation under accession number HRA001009. And the detailed information about single-cell RNA-seq library preparation and sequencing can be got from the unpublished manuscript of Qian-Fei Wang and his colleagues [20]. For downstream analysis, we focused on transcriptionally defined leukemia cells identified by Qianfei Wang et al. [20]. To investigate whether LDB1/LMO2 related to self-renewal function, the leukemia cells from each patients were divided into LSC-high (including those resembling HSC or LMPP) or LSC-low according to the enrichment of LSC signatures [21, 22] using Gene Set Enrichment Analysis (GSEA). Then the expression of LDB1/LMO2 and potential targets of LDB1 (Supplementary Table 3) were evaluated in LSC-high or LSC-low leukemia cells as previously described [23]. Briefly speaking, the up-and downregulated genes identified in LDB1-deficinet cells (Supplementary Table 3) were collected as two signatures. Signature scores were then computed in each cells using the Seurat function “AddModuleScore”. The two scores were then collapsed into one score by taking the difference of the signature scores for up- and downregulated gene signatures to represent the expression of LDB1 targets. The collapsed score was compared between LSC-high and LSC-low leukemia cells.

### In vivo experiments

Based on past experience [24] 4–6 week-old BALB/c female mice were randomly divided into two groups: sh-NC and sh-Ldb1, and each group contains seven mice. The P388D1 cells expressing firefly luciferase (P388D1.ffluc) were transfected with sh-NC or sh-LDB1 and were injected into the tail vein with 2 × 10^5^ cells suspensions in 100ul PBS for each mice . D6/9/12 days after tail vein injection, D-luciferin sodium salt (115144-35-9, GOLDBIO, USA) was injected into the abdominal cavity of tumor-bearing mice, and small animal live imager (Berthold, Germany) scans were performed to measure the maximum standard Avg radiance uptake value of tumors in mice. Animals were anesthetized with 2% isoflurane and set for the acquisition of PET scan within 30 min. Mice were first euthanized by inhalation of CO_2_, and tissues, such as liver, spleen and bones were collected for subsequent experimental procedures, including paraffin block and HE staining. IHC was performed as previously described [25]. Primary antibodies against Ki67(GB12114, Servicebio, China) were used according to the manufacturer’s recommendations.

### Statistical analysis

Experimental results were expressed as mean ± standard error of the mean of multiple experiments or mean ± standard deviation from the representative experiments. All statistical analysis were performed using GraphPad Prism 7.0 software (San Diego, CA, USA).The student’s t test (unpaired, two-tail) was used to assess the significance of the comparative analysis between the two groups, and P < 0.05 was considered statistically significant(*P <0.05,**P <0.01,and ***P <0.001). Survival curve analysis was compared by Log-Rank test, and P <0.05 indicate statistical significance.

## Results

### LMO2 is overexpressed in patients with AML and is associated with poor prognosis

**Fig. 1:**
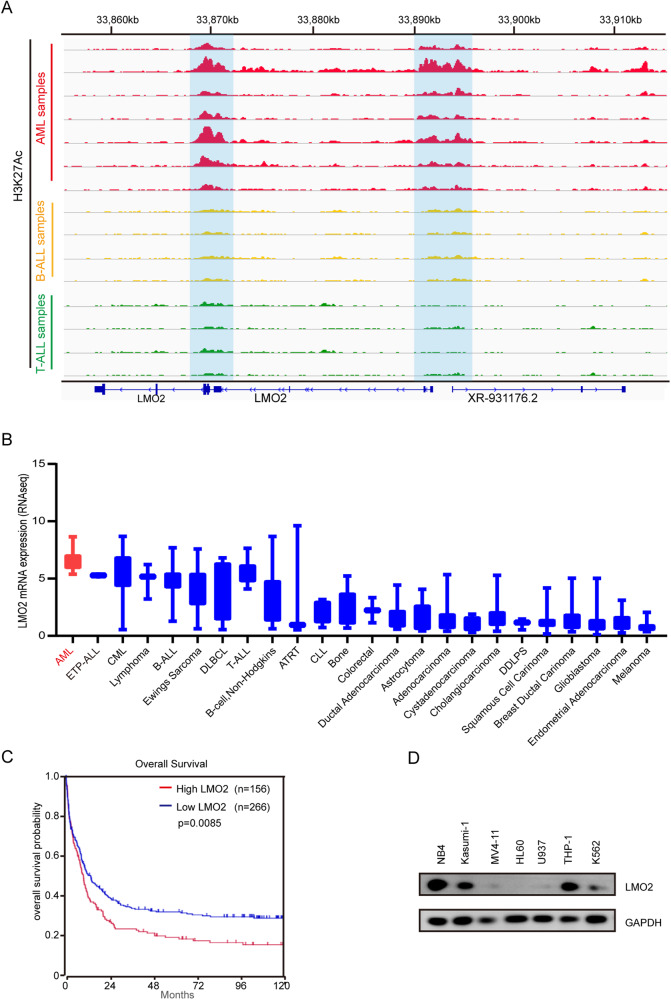
LMO2 is upregulated in AML and is related to prognosis. **A** Using the IGV visualization tool to compare the ChIP-Seq results of clinical samples of children with AML for H3K27Ac, the area near LMO2 was enriched, and there was a high possibility of super-enhancers. **B** Expression levels of LMO2 in common cancer cells in the CCLE database. **C** According to the The Cancer Genome Atlas (TCGA) database, patients with high LMO2 expression in Patients with AML have a lower survival rate. **D** Validated Western-blot data in AML cell lines, suggesting that LMO2 is expressed to varying degrees.

LMO2 is widely recognized as an oncogene. To explore the clinical significance of LMO2 in patients with AML, we performed H3K27ac ChIP-Seq analysis on 7 patients with AML. The IGV visualization tool showed that there was a high binding peak region of H3K27ac in the adjacent region of the LMO2 gene, while the promoter region of the LMO2 gene demonstrated a high binding peak of H3K27ac (Fig. 1A). However, no significant enrichment was found in the LMO2 occupying sites in both B-ALL and T-ALL patients. These results indicate that there may be a super enhancer region near LMO2 in AML cell lines, and the LMO2 gene has strong transcriptional activity.

In order to determine the expression of LMO2 in AML cell lines, we used Cancer Cell Line Encyclopedia (CCLE) database (https://sites.broadinstitute.org/ccle/) to compare the expression of LMO2 in different tumor cell lines. According to the collation of CCLE information, we collected the data of more than 20 tumor types, and arranged each group from high to low in accordance with the expression level of mRNA (Fig. 1B). Results showed that the mRNA expression level of LMO2 ranked first in AML cell lines and second in ETP-ALL, indicating that the LMO2 gene may play an important role in the specific high expression of AML.

Based on the TCGA dataset, 266 AML patients with low expression and 156 AML patients with high expression of LMO2 were divided by median gene expression. Results showed that the prognosis and survival rate of patients with high expression was significantly lower compared to patients with low expression, and the difference was statistically significant (p < 0.01) (Fig. 1C).

Furthermore, AML cell lines were selected and Western-blot was used to detect the protein expression level of LMO2. Results showed that LMO2 was expressed in different degrees in AML cell lines. Among them, the relative expression levels were higher in NB4, Kasumi-1 and K562 cell lines (Fig. 1D).

Overall, our findings demonstrated the high specific expression of the LMO2 gene in AML, which in turn indicate the important pathogenic role of LMO2 in patients of AML.

### LMO2 is required for the survival of AML cell lines

**Fig. 2:**
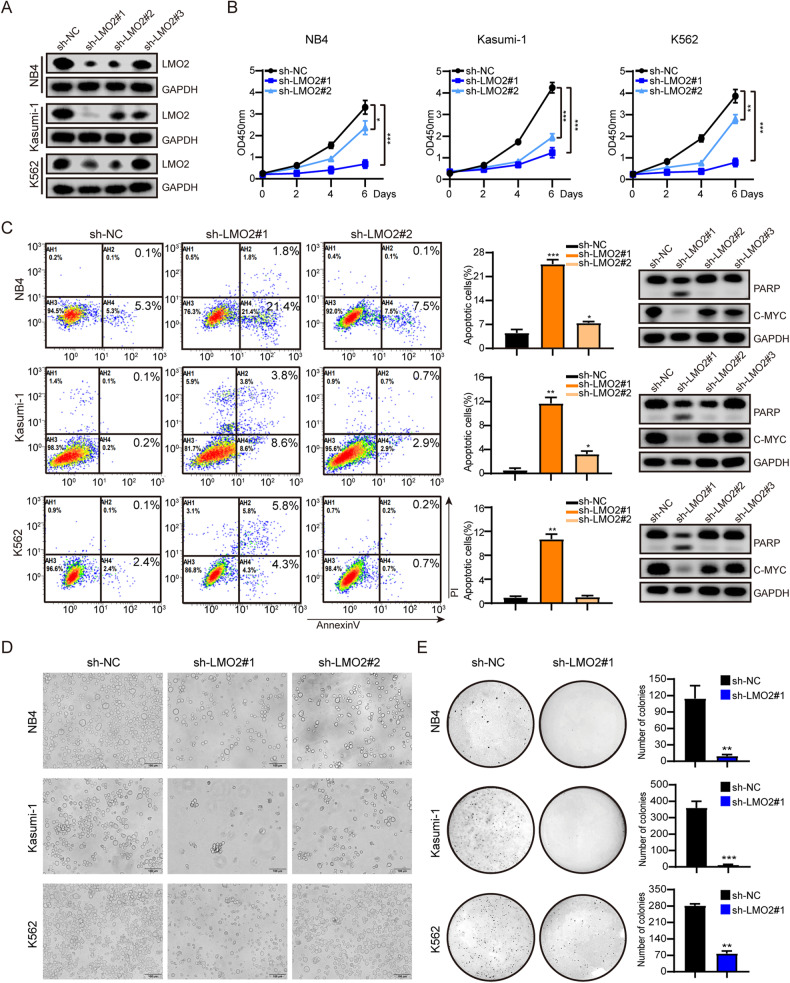
LMO2 knockdown reduces cell proliferation, induce apoptosis, and suppresses the colony-forming capacity in AML cells. **A** LMO2 knockdown in NB4, Kasumi-1, and K562 cells. LMO2 and GAPDH (loading control) protein expression. **B** Cell proliferation was performed by Cell Counting Kit 8 (WST-8/CCK-8) assay over a 4-day period in NB4, Kasumi-1, and K562 cells. **C** Apoptosis by Annexin V staining. (Right) Protein expression levels of PARP, CMYC, and GAPDH (loading control). **D** Microscopic changes of NB4, Kasumi, and K562 cells after LMO2 knockdown. **E** Colony numbers of NB4, Kasumi-1, and K562 cells after LMO2 knockdown in soft agar. Data are presented as mean ± sd. The colony experiments were performed independently twice times, other experiments were performed in triplicate.

To investigate the role of LMO2 in AML, LMO2 was knocked down by shRNAs in NB4, Kasumi-1, and K562 cell lines. Western-blot was used to compare the expression of LMO2 protein in sh-NC, sh-LMO2#1, sh-LMO2#2, and sh-LMO2#3. Results showed that the level of the LMO2 protein in the interference group was significantly lower compared to the level in the sh-NC group, while sh-LMO2#1 had the best interference effect (Fig. 2A). The Real-time quantitative PCR results showed that the relative expression level of LMO2 mRNA in the interference group was significantly lower than that in the sh-NC group, which was consistent with the results of WB (Supplementary Fig. 1A).

We further evaluated the effect of LMO2 on cell proliferation using NB4, Kasumi-1, and K562 cell lines. The absorbance value at 0、2、4、and 6 days was measured. And the results showed that LMO2 knockdown caused significant growth inhibition in the AML cell lines (Fig. 2B). To characterize the apoptotic profile of AML cell lines with LMO2 knockdown, the apoptotic status was assessed by flow cytometry. Knockdown of LMO2 was found to increase the Annexin V+ fraction and promote apoptosis of NB4, Kassumi-1, and K562 cell lines (Fig. 2C). Western blotting revealed that the expression of oncogene c-Myc decreased, while the cleavage band of apoptotic protein PARP was obvious (Fig. 2C). The morphology of the LMO2-knockdown cells was observed under microscope, and results showed that NB4, Kasumi-1, and K562 cell lines in the interference group were all significantly restricted in growth and died compared with the sh-NC group (Fig. 2D).

Furthermore, we performed a colony-forming assay using AML cell lines from sh-NC or sh-LMO2. Knockdown of LMO2 attenuated the colony-replicating activity of NB4, Kasumi-1 and K562 cell lines compared with the control group (Fig. 2E)

These results indicate that LMO2 plays a role in maintaining cell growth and proliferation, thereby promoting the survival of AML cell lines.

### LMO2 interacts directly with LDB1 in AML cell lines [26] and LDB1 is required for cell survival

**Fig. 3:**
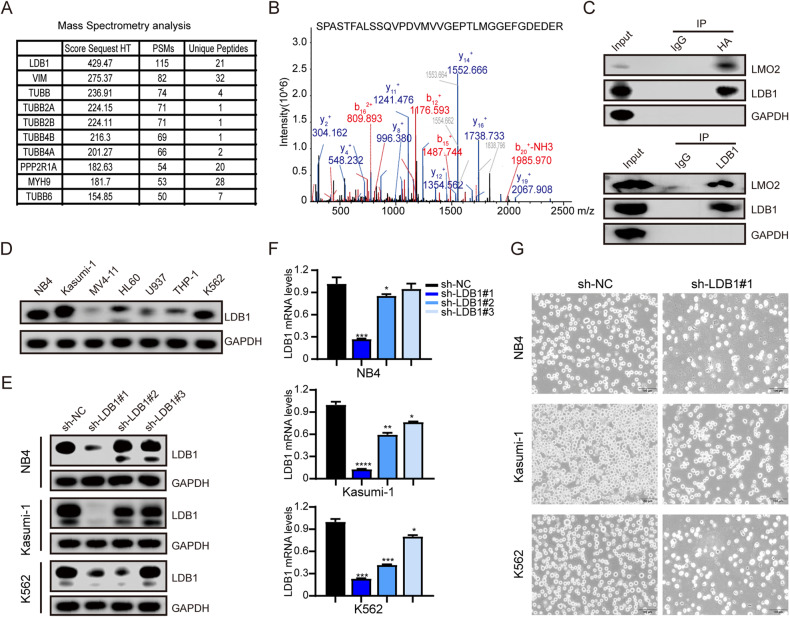
LMO2 directly interacts with LDB1 in AML cells. **A** The proteins identified by mass spectrometry were sorted according to the score of protein matching degree by Sequest HT. LDB1 was in the first place, and the number of peptides matched to the secondary map (PSMs) was also in the forefront. **B** The two-dimensional peak diagram shows one of the sequences of the peptide corresponding to the LDB1 protein. **C** NB4 cells expressing LMO2 were immunoprecipitated with HA antibody, and LDB1 protein was detected by WB. NB4 cells were immunoprecipitated with LDB1 antibody and LMO2 protein was detected by WB. **D** Expression of LDB1 protein in AML cell lines. **E** The LDB1 mRNA was detected by qRT-PCR in shRNA-transfected NB4, Kasumi-1, and K562 cells. **F** LDB1 and GAPDH (loading control) protein expression. **G** Microscopic cell morphology of NB4, Kasumi, and K562 cells after LDB1 interference. Data are presented as mean ± sd for at least three independent experiments.

LMO2 may contribute to cancer development by mediating protein-protein interactions [7]. To identify the proteins interacting with LMO2 in AML, we overexpressed HA-labeled LMO2 in 293 T cells and carried out co-immunoprecipitation. Mass spectrometry identified LIM domain-binding protein 1 (LDB1) as a potential binding protein to LMO2 (Fig. 3A, B, Supplementary Table 2). It has been reported that LDB1 is a necessary partner for LMO2-induced T-ALL and plays an important role in stabilizing the LMO2 transcription factor complex [13].

Based on the prediction of protein interaction information in the biological information database GENEMANIA (http://genemania.org), proteins that may interact with LDB1 were searched. Among the predicted proteins, LMO2 was identified, a protein which plays an important role in AML (Supplementary Fig. 1B).

To verify the binding of LDB1 to LMO2, we verify each other in both positive and negative ways. We performed protein immunoprecipitation experiment on 293 T cells overexpressing LMO2 with HA label. Specific immunoprecipitation of the LMO2 protein complex was performed using HA antibody, and LDB1 protein was detected in the Western-blot detection complex (Fig. 3C). LDB1 antibody was used to immunoprecipitated the LDB1 protein complex in NB4 cells, and LMO2 was detected in the protein complex by WB.

According to the sorting of the CCLE database, LDB1 is ubiquitously expressed in more than 20 tumor cell lines, among which AML cell lines have the higher expression (Supplementary Fig. 1C). Western-blot was used to confirm the expression of LDB1 protein in AML cell lines (Fig. 3D). The results showed that LDB1 was highly expressed in AML cell lines and may play important roles in cell survival and proliferation.

We successfully constructed LDB1 knockdown vector to transfect NB4, Kasumi-1, and K562 cell lines, and verified its expression using RT-PCR and Western-blot (Fig. 3E, F). The results showed that the relative expression levels of LDB1 mRNA and protein in the interference group were significantly lower than those in the sh-NC group, suggesting that lentiviral vectors were effective in silencing the LDB1 gene.

Microscopic observation of cell morphology showed that the growth of the sh-LDB1#1 group of NB4, Kasumi-1, and K562 cells was restricted significantly compared with the sh-NC group, indicating that RNA knockdown produced a considerable effect (Fig. 3G).

These results suggest that LMO2 and LDB1 could form a protein complex, and LDB1 gene knockout can inhibit the growth of AML cells.

### LDB1 is necessary for AML cell survival and colony formation

**Fig. 4:**
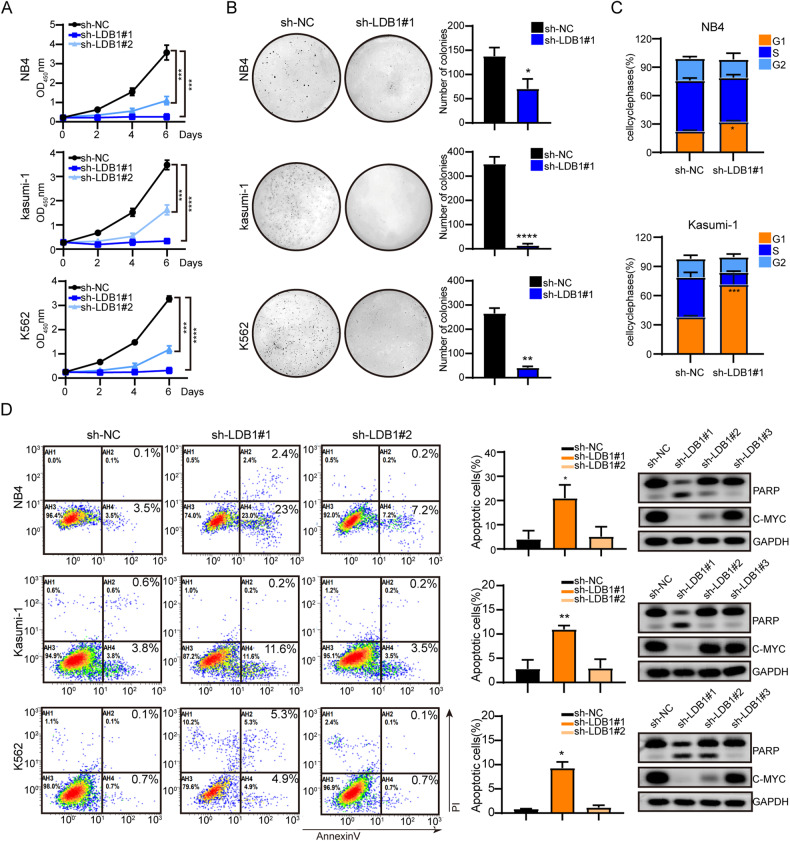
LDB1 knockdown promoted apoptosis, reduced cell proliferation, and suppressed the colony-forming capacity of AML cells. **A** Lower cell viability was measured by CCK-8 assays in NB4, Kasumi-1, and K562 cells transfected with LDB1. **B** Colony formation ability was attenuated by LDB1 transfection. **C** LDB1 knockdown in NB4 and Kasumi-1 cells, cell cycle profile measured by PI staining. **D** The apoptotic rates of transfected NB4, Kasumi-1, and K562 cells were measured by flow cytometry. (Right) Protein expression levels of PARP, CMYC, and GAPDH (loading control). Data are presented as mean ± sd. The colony experiments were performed independently twice times, other experiments were performed in triplicate.

To further evaluate the role of LDB1 in AML cell lines, we examined the effect of LDB1 knockdown on the proliferation of NB4, Kasumi-1, and K562. Results showed that the relative proliferation ability of the sh-LDB1 group was lower than that of the sh-NC group (Fig. 4A). Knockdown of LDB1 reduced the colony-forming ability of NB4, Kasumi-1, and K562 cell lines (Fig. 4B). Meanwhile, we confirmed that cell cycle was arrested in the G0/G1 phase after LDB1 knockdown (Fig. 4C).

Flow cytometry detected the apoptosis of NB4, Kasumi-1, and K562 cell lines after LDB1 knockdown. The apoptosis rate of the sh-LDB1#1 group was significantly higher than that of the sh-NC group, and the difference was statistically significant (Fig. 4D). Consistent with the above findings, the cleavage band of PARP protein was evident and the c-Myc protein was significantly downregulated after LDB1is knockdown (Fig. 4D).

Eventually, the above data indicate that LDB1 knockdown could inhibit the survival of AML cell lines through apoptosis and cell cycle arrest. Furthermore, the ability to form colony was decreased, thereby reducing the tumorigenicity of malignant cells.

### Anti-tumor effect of Ldb1 knockdown in mouse leukemia model

To investigate the impact of Ldb1 on leukemogenesis, Ldb1 was knocked down in mouse P388D1 cells by constructing shRNA vector. We first detected the expression levels of mRNA and protein in P388D1.ffluc cells transfected with sh-NC or sh-Ldb1. Results showed that the relative mRNA expression and protein level of Ldb1 in the interference group were significantly lower compared to the sh-NC group (Supplementary Fig. 2A). While, sh-Ldb1 #2 has the best interference effect. The CCK-8 experiment revealed that the proliferation ability of P388D1.ffluc cells in the sh-Ldb1#2 group was lower than that in the sh-NC group (Supplementary Fig. 2B).

**Fig. 5:**
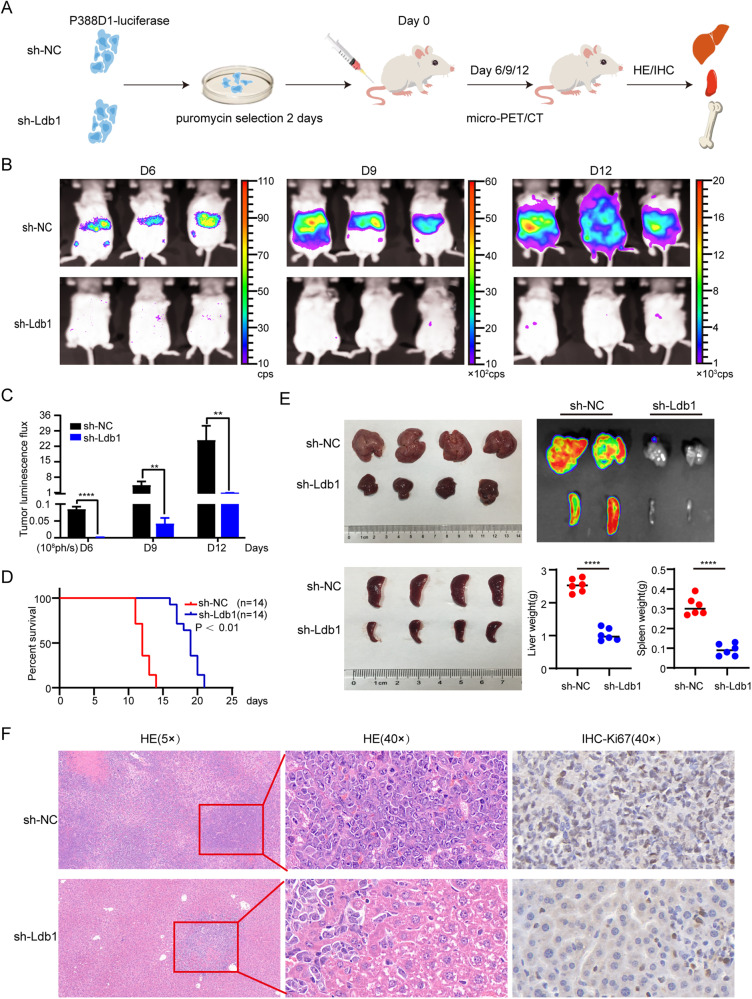
Ldb1 displays anti-tumor efficacy in a mouse leukemia model. **A** Schematic diagram of experimental design. **B** Representative bioluminescent images of mice treated with Ldb1 knockdown P388D1.ffluc cells or sh-NC-P388D1.ffluc cells. **C** Comparison of the maximum standard Avg radiance uptake value. **D** Female sh-Ldb1-treated mice had significantly prolonged survival compared with the controls. **E** Different sizes and weights of liver and spleen, from sh-NC or sh-Ldb1 mices. Biological image of Avg radiance uptake by liver and spleen tissue from BALB/c mice. **F** Representative images of HE staining and IHC staining of BALB/c mice livers.

Female BALB/c mice were divided into the sh-NC and sh-Ldb1 groups, and injected with sh-NC-P388D1.ffluc cells or sh-Ldb1-P388D1.ffluc cells via tail vein, respectively. Comparisons between the two groups were then performed by observing indicators, such as survival time, in vivo imaging of small animals, tumor volume, and immunohistochemistry (Fig. 5A).

In vivo imaging of small animals showed that fluorescence intake in the Ldb1 knockdown group was lower than that in the control group, suggesting that tumor burden was reduced after Ldb1 knockdown (Fig. 5B, C). A significant median survival advantage was found in Ldb1 knockdown mice by survival follow-up (Fig. 5D).

Moreover, the liver and spleen of mice in the sh-NC group were larger than those in the sh-Ldb1 knockdown group (Fig. 5E). More specifically, ex vivo bioluminescence imaging revealed that the main internal organs through which P388D1.lus cells spread were the liver and spleen.

HE results confirmed the histological evidence of P388D1.lus cells infiltration in the liver and spleen (Fig. 5F and Fig. S2C). IHC staining of Ki67 in liver and spleen tissues confirmed that the knockdown of Ldb1 has an inhibitory effect on the development of tumor (Fig. 5F, Supplementary Fig. 2C).

In conclusion, there is consistency between the results of the in vivo and in vitro experiments, which together prove that Ldb1 knockdown has an anti-tumor effect in leukemia.

### Exploring potential targets of LDB1 in AML

**Fig. 6:**
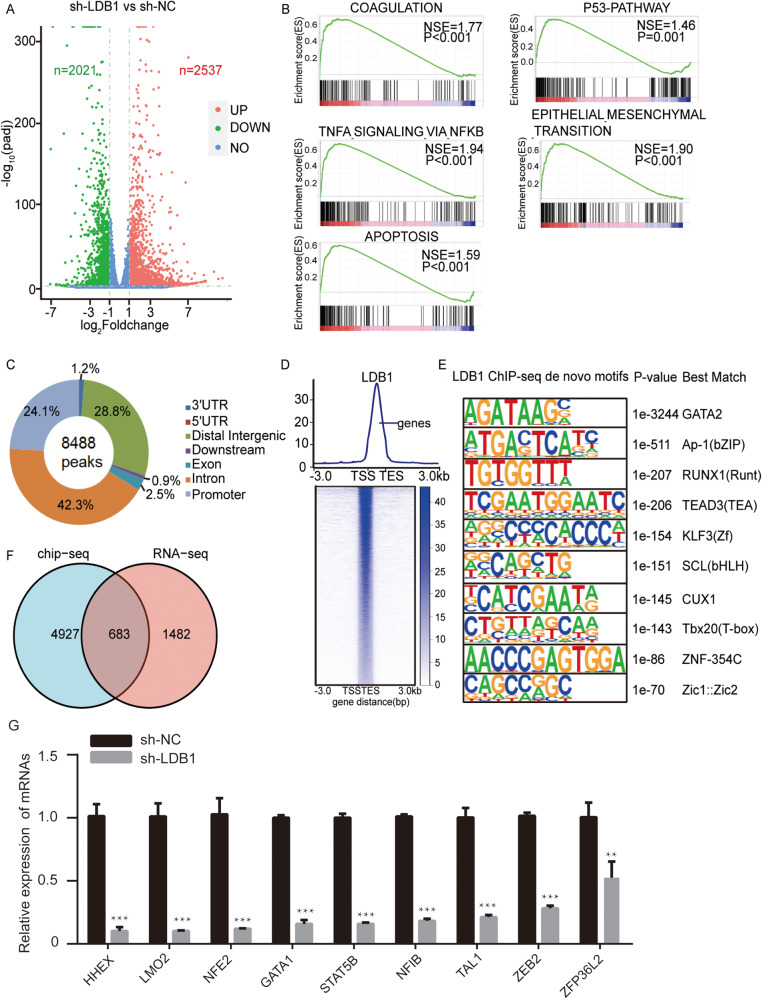
Transcriptomic identification of potential targets of LDB1 in acute myeloid leukemia cells. **A** RNA seq volcano plots showing changes in gene expression following LDB1 knockdown in NB4 cells. Red and blue indicate upregulated and downregulated genes, respectively. **B** GSEA analysis revealed the most abundant gene expression signatures in NB4 cells after LDB1 deletion. **C** Distribution of LDB1 binding to genomic regions in the NB4 cells, as assessed by ChIP-Seq. The pie chart showing the peak annotation of LDB1. **D** Heat map, analyzed from ChIP-Seq data, showing the occupancy of LDB1 in NB4 unit. Centering on LDB1 peak, this is further divided into TSS and non-TSS regions. **E** According to the ChIP-Seq, HOMER de novo motif analysis was then performed on the sequence data of LDB1 ChIP. The identified de novo motif was matched to a database of known motifs. **F** Comprehensive analysis for the identification of transcriptome-wide potential targets of LDB1 in acute myeloid leukemia by RNA-seq and ChIP-Seq results. **G** Validation of mRNA expression changes in potential gene targets of LDB1 by RT-PCR.

To investigate the underlying molecular mechanisms by which LDB1 functions in AML, RNA-seq analysis was performed on human NB4 cells with LDB1 knockdown (GSE213913). We introduced the lentiviral vector sh-LDB1 and the control vector sh-NC into NB4 cells, and then extracted total RNA from the cells for RNA-seq analysis. 2537 upregulated and 2021 downregulated genes were identified in the LDB1-knockdown cells (Fig. 6A, Supplementary Table 3). Notably, 27 genes that are associated with hematopoietic stem cell self-renewal were observed to be downregulated (Supplementary Table 4). We further investigated the expression of LDB1 and its putative target genes in leukemia stem cells derived from AML patients, utilizing publicly available single-cell RNA sequencing (scRNA-seq) data obtained from AML patient [20]. Based on the expression of well-established leukemia stem cell signatures [21, 22], leukemia cells from each patient into LSC-high or LSC-low groups and the expression of LDB1 target genes were compared (Supplementary Fig. 3). Building upon previous observation that LSC-high cells resembling HSC or LMPP populations were identified in 11 AML patients [20], we found that LSC-high cells resembling the HSC population exhibited higher expression levels of LDB1 targets compared with LSC-low cells in 5 out of 7 patients. However, LSC-high cells resembling LMPP population showed lower expression of LDB1 targets compared with LSC-low cells in all of the 6 patients. These findings collectively support the notion that LDB1 may have a role in HSC self-renewal process in leukemia.

Gene concentration analysis (GSEA) revealed that genes related to leukemia apoptosis and tumor necrosis factor were more abundant in LDB1 knockdown cells than in the control cells (Fig. 6B). We next assessed the binding pattern of LDB1 across genomic regions using ChIP-Seq in the NB4 cell line (GSE213636), and found that LDB1 functions cooperatively with GATA2, RUNX1, TEAD3, KLF3, SCL, CUX1, Tbx20, and ZNF-354C (Figs. 6C–E, Supplementary Table 5). We have performed NB4 LDB1 CUT&Tag (GSE228147), and the results of NB4 LDB1 ChIP-Seq and NB4 LDB1 CUT&Tag are fairly consistent (Supplementary Fig. 4). We observed 1101 overlaps between LDB1-binding genes in ChIP-Seq results and abnormally expressed genes in RNA-seq with LDB1 knockdown, including LMO2 (Fig. 6F, Supplementary Table 6). The qPCR results confirmed that the expression levels of these genes were consistent with the sequencing results in AML cells after LDB1 knockdown (Fig. 6G).

**Fig. 7:**
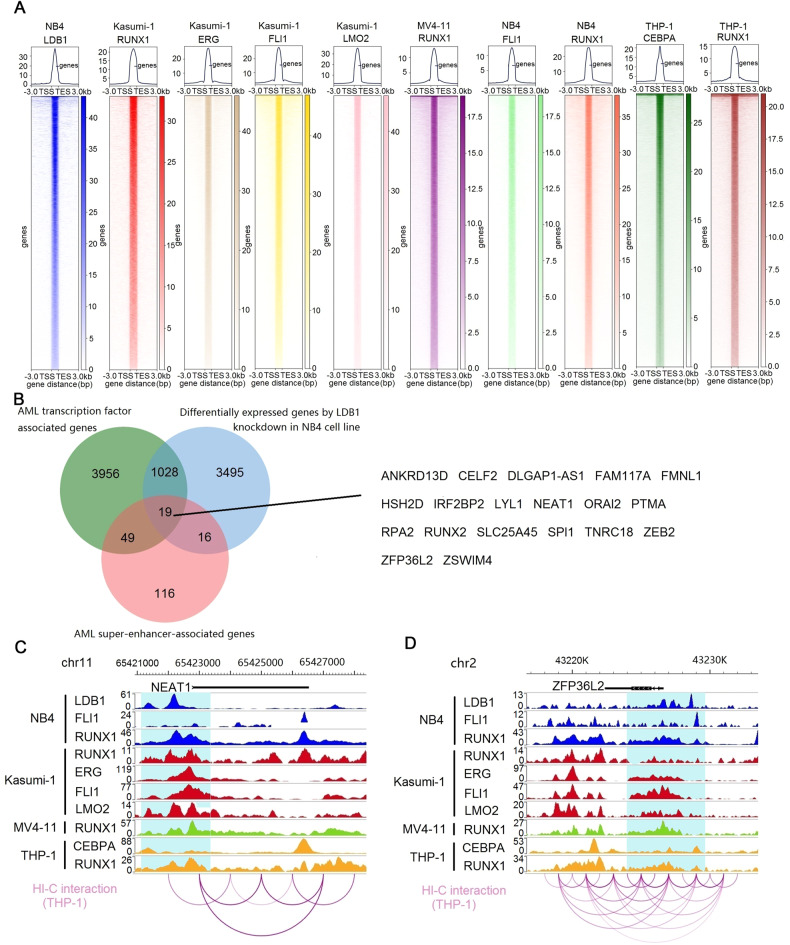
Prediction the potential targets of LDB1 in AML. **A** Heatmaps generated from ChIP-Seq data analyses showed the occupancy of RUNX1, ERG, FLI1, and LMO2 in Kasumi-1 cell line, RUNX1 in MV4-11 cell line, FLI1 and RANX1 in NB4 cell line, CEBPA, and RUNX1 in THP-1 cell line. **B** Venn diagram of genes associated with AML transcription factors, super-enhancers, and sensitive to LDB1 knockdown in AML cells. **C** The results of 10 ChIP-Seq datasets for LDB1, RUNX1, ERG, FLI1, LMO2, and CEBPA in AML cell lines showed that those transcription factors bound to NEAT1 (track 1-10), and the Hi-C data analysis of THP-1 cell line (track 11) represents interaction at the NEAT1 gene loci. **D** The results of 10 ChIP-Seq datasets for LDB1, RUNX1, ERG, FLI1, LMO2, and CEBPA in AML cell lines showed that those transcription factors bound to ZFP36L2 (track 1-10), and the Hi-C data analysis of THP-1 cell line (track 11) represents interaction at the ZFP36L2 gene loci.

We also analyzed the public ChIP-Seq datasets of RUNX1, ERG, FLI1, and LMO2 in Kasumi-1 cell line, RUNX1 in MV4-11 cell line, FLI1 and RUNX1 in NB4 cell line, CEBPA and RUNX1 in THP-1 cell line. The results of the total 10 ChIP-Seq datasets for LDB1, RUNX1, ERG, FLI1, LMO2, and CEBPA in AML cell lines showed that those transcription factors bound to 5052 genes in ≧8 ChIP-Seq datasets (Fig. 7A, B, Supplementary Table 7). We next performed filtering to identify genes that were also significantly abnormally expressed after LDB1 knockdown (P < 0.05 and fold-change >2 or fold-change <0.5) in NB4 cells (Fig. 7B). Additionally, we previously performed H3K27ac ChIP-Seq in AML samples [27], and performed filtering to identify genes that also harbor super-enhancers in ≧10 AML samples (Fig. 7B, Supplementary Table 8). Filtering according to the above stringent criteria narrowed down the list of candidates to 19 genes, including IRF2BP2, LYL1, NEAT1, and ZFP36L2 (Fig. 7B, Supplementary Table 9). The ChIP-Seq data for LDB1, RUNX1, ERG, FLI1, LMO2, and CEBPA in AML cell lines showed that the gene region of IRF2BP2, LYL1, NEAT1, and ZFP36L2 had strong signals (Fig. 7C, track 1-10, Fig. 7D, track 1-10, Supplementary Fig. 5, track 1-10, Supplementary Fig. 6, track 1-10). The results of Hi-C data also represent interaction in the gene region of IRF2BP2, LYL1, NEAT1, and ZFP36L2 in THP-1 cell line (Fig. 7C, track 11, Fig. 7D, track 11, Supplementary Fig. 4, track 11, Supplementary Fig. 5, track 11).

### LMO2/LDB1 is required for proliferation of primary AML cells, and over expression of LMO2 partially compensates the functional loss of LDB1 in AML cell lines

**Fig. 8:**
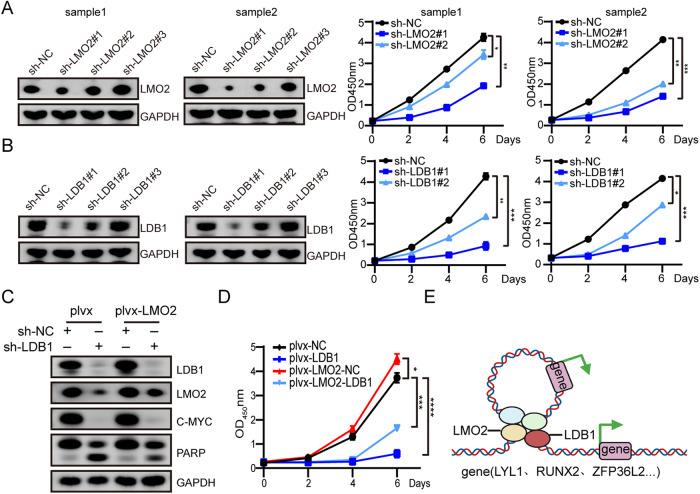
LMO2/LDB1 is required for proliferation of primary AML cells, and LMO2 partially mediates LDB1 function in AML cells. **A** LMO2 knockdown in primary AML cells. LMO2 and GAPDH (loading control) protein expression (left). cell proliferation was performed by CCK-8 assay (right). **B** LDB1 knockdown in primary AML cells resulted in reduced cell proliferation ability. The representative results from 2 AML patients as show. **C** LMO2-overexpressing NB4 cells (plvx-LMO2) and controls (plvx) were transfected with sh-NC and sh-LDB1, respectively. LDB1, LMO2, PARP, c-Myc, and GAPDH protein expression. **D** Cell growth of plvx and plvx-LMO2 NB4 cells transfected with sh-NC and sh-LDB1. **E** Schematic representation of LDB1 in the pathogenesis of AML. In AML, LMO2 can form a protein complex with LDB1. LDB1 knockdown can target the expression of LYL1, RUNX2, and other genes to regulate the malignant phenotype of AML by promoting apoptosis and inhibiting proliferation. Data are presented as mean ± sd for twice independent experiments.

In order to investigate the effect of LMO2 on AML primary cells, we knocked down LMO2 in primary AML cells and decreased the ability of cell proliferation (Fig. 8A). Knockdown of LDB1 also reduced the growth of primary AML cells (Fig. 8B).

To further identify the interaction between LMO2 and LDB1, we constructed NB4 cell lines overexpressing LMO2 or knocking down LDB1. After LDB1 knockdown, LMO2 expression was lower than that in the sh-NC group. The LDB1 expression level in cells overexpressing LMO2 was higher than that in cells without LMO2 overexpression (Fig. 8C). Consistently, the overexpressing LMO2 in NB4 cells with LDB1 knockdown, c-Myc protein expression was partially restored and the PARP cleavage band was reduced. Furthermore, the reintroduction of LMO2 was found promote cell growth of NB4 cells (Fig. 8D).

In the context of AML, LMO2 can form a protein complex with LDB1, functioning as a super enhancer. By downregulating LDB1, the expression of LYL1, RUNX2, and other genes can be targeted, thereby regulating the malignant phenotype of AML through the promotion of apoptosis and inhibition of proliferation (Fig. 8E). Our results show that LDB1 and LMO2 could function as oncogenes in AML cells through a mutually regulated manner, which could be used as a potential target for clinical treatment of patients with AML.

## Discussion

Increasingly more and more evidence showed that novel targeted drugs are a promising strategy for the treatment of leukemia, hence, it is urgent to identify new molecular targets for the development of effective therapeutic methods for AML [3]. The function of LMO2 varies greatly in different tumors. It has been reported that LMO2 played a carcinogenic role in prostate cancer and glioblastoma [28, 29], and yet is a good prognostic marker for diffuse large B-cell lymphoma and pancreatic cancer [30, 31]. These opposite functions may be due to the fact that LMO2 can regulate different cellular signaling pathways by mediating protein-protein interactions in different tumors [7]. Research has demonstrated that Lmo2 is capable of promoting the growth of self-renewing preleukemic stem cells (pre-LSCs) and the onset of ETP-ALL [32]. Upon scrutinizing the CCLE database, it was discovered that LMO2 is markedly expressed in ETP-ALL, yet more so in AML, thereby suggesting that LMO2 is selectively overexpressed in AML and may hold significant significance. Our results showed that LMO2 knockdown significantly inhibited the proliferation of AML cells and promoted apoptosis. It has also been confirmed that LMO2 knockout could damage the growth of MLL-enl cells. This is consistent with our findings. Among AML patients with normal karyotype, those with high expression of LMO2 have a poor prognosis [33].

Meanwhile, the functions of LMO2 in a certain cell type depends not only on the abundance of LMO2 itself, but also on its preferred partners [6]. LMO2 could regulate the expression of oncogenes and cell differentiation of AML cell lines through complex formation with MYB [34]. The interaction between LMO2 and transcription factor Zbtb1 is necessary to maintain the T-lineage differentiation capacity of lymphoid progenitor cells [35]. Mass spectrometric analysis showed that LDB1 ranked first among the proteins interacting with LMO2 in AML cells. There are other proteins, such as GATA1, ISL1 and PTEN, which are key factors involved in the regulation of multiple cell activities. We do not rule out the interaction between LMO2 and other protein factors. The interaction between transcription factor LDB1 and LMO2 has also been reported. The nuclear adapter Ldb1 is required for Lmo2 oncogene-induced thymocyte self-renewal and T-cell leukemia in a mouse model of T-ALL [13].

Transcription factor LDB1 is also involved in a variety of cellular activities, including tumors. In the regulation of erythrocyte transcription, LDB1 is required for the activation of erythrocyte genes, including globin genes, while in myeloid cells, LDB1 can promote and stabilize the binding of PU.1 to enhancer URE in AML cells [36]. The absence of LDB1 could reduce the invasion and migration in oral cancer cell lines [37]. In addition, the overall survival rate and metastasis-free survival rate was reduced in patients with high LDB1 expression [38, 39]. LDB1 could bind to a variety of transcription factors, and play a regulatory role in the form of active complexes, including DNA binding transcription factors GATA1, TAL1, and scaffold protein LMO2 [24]. In our study we confirmed that LMO2 and LDB1 combination plays oncogenes role in AML. Our results showed that LDB1 knockout significantly inhibited cell proliferation and promoted apoptosis. In the mouse model, Ldb1 knockdown significantly inhibited tumor formation and prolonged the survival time. Meanwhile, knockdown of LMO2 and LDB1 reduced the proliferation of primary AML cells in vitro. These findings further support our data that LDB1 is a negative prognostic factor for AML. Transcriptome sequencing found that a variety of signaling pathways were regulated, such as COAGULATION, p53-pathway, TNFA signaling VIA NFKB and APOPTOSIS after LDB1 was knocked down. We speculate that LMO2/LDB1 is involved in the regulation of a variety of signaling pathways, thus promoting the development of AML.

According to reports, Ldb1, a fundamental constituent of the transcriptional complex, plays a pivotal role in regulating the transcriptional process that maintains fetal and adult mouse hematopoietic stem cells (hsc) [40]. The outcomes of single-cell sequencing revealed that HHEX, NFE2, ZFP36L2, and other genes exhibited high expression levels in LSC-high patients. Notably, The RNA-seq findings reveal a significant down-regulation of genes linked to hematopoietic stem cell self-renewal, namely HHEX and NFE2, upon LDB1 knockdown. Prior research has established HHEX’s role in regulating hematopoietic stem cell self-renewal and the onset and persistence of acute myeloid leukemia [41]. Additionally, NFE2 serves as a crucial driver of human hematopoietic stem cell maintenance and T lineage differentiation, and can facilitate the progression of AML [42, 43]. The aforementioned findings indicate that the modulation of genes associated with hematopoietic stem cell (HSC) self-renewal may serve as a contributing factor in the progression of acute myeloid leukemia (AML) via LDB1. Unfortunately, we have not carried out the research to explore the function of protein interactions, which needs to be further studied.

Interestingly, LMO2 is also present in the downstream genes regulated by LDB1, and can partially restore the function of LDB1 after reintroduction of LMO2. This indicates that LDB1 and LMO2 can regulate each other and play a carcinogenic role in AML cell lines. Similar to the erythroid genes, proteomics have revealed the existence of an LDB1/LMO2 complex in myeloid cell lines [34]. Some studies have also shown that the LDB1/LMO2 complex is one of the driving factors of leukemia. Furthermore, LDB1 is a necessary partner of LMO2, especially when it comes to the T-ALL mouse model, confirming that Ldb1 plays a role in Lmo2-induced leukemia [13]. LDB1 can stabilize direct and indirect binding partners and is the main core subunit that may be targeted in leukemia. Moreover, the steady-state abundance of LMO2 increases with co-expression of LDB1; hence, targeting LDB1/LMO2 becomes a treatment possibility for leukemia [44]. This is consistent with our research results, which prove that LDB1 and LMO2 are mutually regulated. Cells expressing LMO2 in DLBCL and TAL are highly sensitive to PARP inhibitors, which have a potential therapeutic effect on LMO2 positive tumors [45]. It has been also confirmed that NAMPT can activate LMO2 through lysine deacetylation, which further promotes LMO2 and LDB1 interactions and forms a transcriptional active TAL1 complex. Specific NAMPT inhibitors, such as FK866, are expected to be one of the targeted therapies for T-ALL [46]. These targeted drugs are considered potent and novel strategies for the treatment of AML.

## Conclusion

To summarize, this study demonstrates that LDB1 is a novel regulatory factor of leukemia. LDB1 could promote leukemia development by regulating LMO2 expression, and forming the LDB1/LMO2 protein complex in AML. Blocking the formation of the LDB1/LMO2 complex represents a novel therapeutic strategy for the treatment of leukemia. Thus, we provided compelling evidence demonstrating that the LDB1 contributes to tumorigenesis and poor prognosis in AML and can serve as a target for the development of cancer therapeutics.

## Supplementary information

Supplementary Figure and Table Legends

Supplementary Figure 1

Supplementary Figure 2

Supplementary Figure 3

Supplementary Figure 4

Supplementary Figure 5

Supplementary Figure 6

Supplementary Table 1

Supplementary Table 2

Supplementary Table 3

Supplementary Table 4

Supplementary Table 5

Supplementary Table 6

Supplementary Table 7

Supplementary Table 8

Supplementary Table 9

Supplementary Table 10

Original Data File

checklist

## Data Availability

The data that support the findings of this study are available on reasonable request from the corresponding author. LDB1 RNA‑seq, ChIP-Seq, and CUT&Tag data have been submitted to the GEO database with Accession Number GSE213913, GSE213636, and GSE228147, respectively. Processed scRNA-seq data of bone marrow aspirates of thirteen AML patients were obtained from Genome Sequence Archive for Human at the BIG data center, Beijing Institute of Genomics, Chinese Academy of Sciences, and China National Center for Bioinformation under accession number HRA001009.

## References

[1] Récher C (2021). Clinical implications of inflammation in acute myeloid leukemia. Front Oncol.

[2] Pasquer H, Tostain M, Kaci N, Roux B, Benajiba L (2021). Descriptive and functional genomics in acute myeloid leukemia (AML): paving the road for a cure. Cancers (Basel).

[3] Vagapova ER, Lebedev TD, Tikhonova AD, Goikhman BV, Ivanenko KA, Spirin PV (2020). High expression level of SP1, CSF1R, and PAK1 correlates with sensitivity of leukemia cells to the antibiotic mithramycin. Mol Biol (Mosk).

[4] Ptasinska A, Assi SA, Martinez-Soria N, Imperato MR, Piper J, Cauchy P (2014). Identification of a dynamic core transcriptional network in t(8;21) AML that regulates differentiation block and self-renewal. Cell Rep.

[5] Sun XJ, Wang Z, Wang L, Jiang Y, Kost N, Soong TD (2013). A stable transcription factor complex nucleated by oligomeric AML1-ETO controls leukaemogenesis. Nature.

[6] Liu Y, Wang Z, Huang D, Wu C, Li H, Zhang X (2017). LMO2 promotes tumor cell invasion and metastasis in basal-type breast cancer by altering actin cytoskeleton remodeling. Oncotarget.

[7] Wang W, Chen Y, Chang Y, Sun W (2020). Biochemical feature of LMO2 interactome and LMO2 function prospect. Med Sci Monit Basic Res.

[8] Rahman S, Magnussen M, León TE, Farah N, Li Z, Abraham BJ (2017). Activation of the LMO2 oncogene through a somatically acquired neomorphic promoter in T-cell acute lymphoblastic leukemia. Blood.

[9] Goossens S, Wang J, Tremblay CS, De Medts J, T’Sas S, Nguyen T (2019). ZEB2 and LMO2 drive immature T-cell lymphoblastic leukemia via distinct oncogenic mechanisms. Haematologica.

[10] Foran JM (2010). New prognostic markers in acute myeloid leukemia: perspective from the clinic. Hematol Am Soc Hematol Educ Program.

[11] Ohmori S, Ishijima Y, Numata S, Takahashi M, Sekita M, Sato T (2019). GATA2 and PU.1 collaborate to activate the expression of the mouse Ms4a2 gene, encoding FcεRIβ, through distinct mechanisms. Mol Cell Biol.

[12] Yasuoka Y, Taira M (2021). LIM homeodomain proteins and associated partners: then and now. Curr Top Dev Biol.

[13] Li L, Mitra A, Cui K, Zhao B, Choi S, Lee JY (2020). Ldb1 is required for Lmo2 oncogene-induced thymocyte self-renewal and T-cell acute lymphoblastic leukemia. Blood.

[14] Yu X, Martella A, Kolovos P, Stevens M, Stadhouders R, Grosveld FG (2020). The dynamic emergence of GATA1 complexes identified in in vitro embryonic stem cell differentiation and in vivo mouse fetal liver. Haematologica.

[15] Langmead B, Salzberg SL (2012). Fast gapped-read alignment with Bowtie 2. Nat Methods.

[16] Zhang Y, Liu T, Meyer CA, Eeckhoute J, Johnson DS, Bernstein BE (2008). Model-based analysis of ChIP-Seq (MACS). Genome Biol.

[17] Servant N, Varoquaux N, Lajoie BR, Viara E, Chen CJ, Vert JP (2015). HiC-Pro: an optimized and flexible pipeline for Hi-C data processing. Genome Biol.

[18] Yin Y, Yang X, Wu S, Ding X, Zhu H, Long X (2022). Jmjd1c demethylates STAT3 to restrain plasma cell differentiation and rheumatoid arthritis. Nat Immunol.

[19] Robinson JT, Thorvaldsdóttir H, Winckler W, cGuttman M, Lander ES, Getz G (2011). Integrative genomics viewer. Nat Biotechnol.

[20] Zhang Y, Jiang S, He F, et al. Single-cell transcriptomics reveals multiple chemoresistant properties in leukemic stem and progenitor cells in pediatric AML. *International Precision Medicine Conference*, 4th edn (Magnus Group LLC, 2023). 10.1186/s13059-023-03031-7 PMC10472599 37653425

[21] Ross ME, Mahfouz R, Onciu M, Liu HC, Zhou X, Song G (2004). Gene expression profiling of pediatric acute myelogenous leukemia. Blood.

[22] Ng SW, Mitchell A, Kennedy JA, Chen WC, McLeod J, Ibrahimova N (2016). A 17-gene stemness score for rapid determination of risk in acute leukaemia. Nature.

[23] Duy C, Li M, Teater M, Meydan C, Garrett-Bakelman FE, Lee TC (2021). Chemotherapy induces senescence-like resilient cells capable of initiating AML recurrence. Cancer Discov.

[24] Li M, Wei Y, Liu Y, Wei J, Zhou X, Duan Y (2023). BRD7 inhibits enhancer activity and expression of BIRC2 to suppress tumor growth and metastasis in nasopharyngeal carcinoma. Cell Death Dis.

[25] Song D, Guo M, Xu S, Song X, Bai B, Li Z (2021). HSP90-dependent PUS7 overexpression facilitates the metastasis of colorectal cancer cells by regulating LASP1 abundance. J Exp Clin Cancer Res.

[26] Lee J, Krivega I, Dale RK, Dean A (2017). The LDB1 complex co-opts CTCF for erythroid lineage-specific long-range enhancer interactions. Cell Rep.

[27] Fang F, Lu J, Sang X, Tao YF, Wang JW, Zhang ZM (2022). Super-enhancer profiling identifies novel critical and targetable cancer survival gene LYL1 in pediatric acute myeloid leukemia. J Exp Clin Cancer Res.

[28] Chen L, Wang YY, Li D, Wang C, Wang SY, Shao SH (2021). LMO2 upregulation due to AR deactivation in cancer-associated fibroblasts induces non-cell-autonomous growth of prostate cancer after androgen deprivation. Cancer Lett.

[29] Ma S, Guan XY, Beh PS, Wong KY, Chan YP, Yuen HF (2007). The significance of LMO2 expression in the progression of prostate cancer. J Pathol.

[30] Sun C, Cheng X, Wang C, Wang X, Xia B, Zhang Y (2019). Gene expression profiles analysis identifies a novel two-gene signature to predict overall survival in diffuse large B-cell lymphoma. Biosci Rep.

[31] Nakata K, Ohuchida K, Nagai E, Hayashi A, Miyasaka Y, Kayashima T (2009). LMO2 is a novel predictive marker for a better prognosis in pancreatic cancer. Neoplasia.

[32] Latchmansingh KA, Wang X, Verdun RE, Marques-Piubelli ML, Vega F, You MJ (2022). LMO2 expression is frequent in T-lymphoblastic leukemia and correlates with survival, regardless of T-cell stage. Mod Pathol.

[33] Calero-Nieto FJ, Joshi A, Bonadies N, Kinston S, Chan WI, Gudgin E (2013). HOX-mediated LMO2 expression in embryonic mesoderm is recapitulated in acute leukaemias. Oncogene.

[34] Takao S, Forbes L, Uni M, Cheng S, Pineda JMB, Tarumoto Y (2021). Convergent organization of aberrant MYB complex controls oncogenic gene expression in acute myeloid leukemia. Elife.

[35] Koizumi M, Kama Y, Hirano KI, Endo Y, Tanaka T, Hozumi K (2022). Transcription factor Zbtb1 interacts with bridging factor Lmo2 and maintains the T-lineage differentiation capacity of lymphoid progenitor cells. J Biol Chem.

[36] Dean A (2018). PU.1 chromosomal dynamics are linked to LDB1. Blood.

[37] Simonik EA, Cai Y, Kimmelshue KN, Brantley-Sieders DM, Loomans HA, Andl CD (2016). LIM-only protein 4 (LMO4) and LIM domain binding protein 1 (LDB1) promote growth and metastasis of human head and neck cancer (LMO4 and LDB1 in head and neck cancer). PLoS ONE.

[38] García SA, Swiersy A, Radhakrishnan P, Branchi V, Kanth Nanduri L, Győrffy B (2016). LDB1 overexpression is a negative prognostic factor in colorectal cancer. Oncotarget.

[39] Zhu M, Jiang B, Zuo H, Wang X, Ge H, Huang Z (2022). LIM-domain-binding protein 1 mediates cell proliferation and drug resistance in colorectal cancer. Front Surg.

[40] Li L, Jothi R, Cui K, Lee JY, Cohen T, Gorivodsky M (2011). Nuclear adaptor Ldb1 regulates a transcriptional program essential for the maintenance of hematopoietic stem cells. Nat Immunol.

[41] Jackson JT, O’Donnell K, Light A, Goh W, Huntington ND, Tarlinton DM (2020). Hhex regulates murine lymphoid progenitor survival independently of Stat5 and Cdkn2a. Eur J Immunol.

[42] Di Tullio A, Passaro D, Rouault-Pierre K, Purewal S, Bonnet D (2017). Nuclear factor erythroid 2 regulates human HSC self-renewal and T cell differentiation by preventing NOTCH1 activation. Stem Cell Rep.

[43] Jutzi JS, Basu T, Pellmann M, Kaiser S, Steinemann D, Sanders MA (2019). Altered NFE2 activity predisposes to leukemic transformation and myelosarcoma with AML-specific aberrations. Blood.

[44] Layer JH, Christy M, Placek L, Unutmaz D, Guo Y, Davé UP (2020). LDB1 enforces stability on direct and indirect oncoprotein partners in leukemia. Mol Cell Biol.

[45] Parvin S, Ramirez-Labrada A, Aumann S, Lu X, Weich N, Santiago G (2019). LMO2 confers synthetic lethality to PARP inhibition in DLBCL. Cancer Cell.

[46] Morishima T, Krahl AC, Nasri M, Xu Y, Aghaallaei N, Findik B (2019). LMO2 activation by deacetylation is indispensable for hematopoiesis and T-ALL leukemogenesis. Blood.

