# Supplementary material for: LMO2 promotes the development of AML through interaction with transcription co-regulator LDB1
Source: Cell Death Dis. 2023 Aug 12;14(8):518. doi: 10.1038/s41419-023-06039-w (PMC10423285; doi:10.1038/s41419-023-06039-w)
Supplement: Supplementary file 1 — Supplementary Figure and Table Legends [file 41419_2023_6039_MOESM1_ESM.pdf]

Supplementary Figure 1. mRNA expression in AML cells after LMO2 knockdown and prediction of LDB1 potential binding proteins from GENEMANIA database

(A) The LMO2 mRNA was detected by qRT-PCR in shRNA-transfected NB4, Kasumi-1 and K562 cells. Data are presented as mean ± sd for at least three independent experiments.

(B) The GENEMANIA database predicted LMO2 protein among the potential binding proteins of LDB1.

(C)Expression levels of LDB1 mRNA in common cancer cell lines from CCLE database.

Supplementary Figure 2. The knockdown of Ldb1 inhibited the growth of P388D1 cells.

(A) (Left) The relative expression level of Ldb1 Mrna. (Right) The expression level of Ldb1 protein.

(B) After transfection of P388D1.ffluc cells with sh-Ldb1, cell viability decreased. Data are presented as mean ±sd for at least twice independent experiments.

(C) Representative images of HE staining and IHC staining of BALB/c mice spleen.

Supplementary Figure 3. The LDB1 targets were highly expressed in LSC cells resembling HSC in scRNA-seq of AML patients.

Supplementary Figure 4. The results of NB4 LDB1 CHIP-Seq and NB4 LDB1 CUT&Tag datasets.

Supplementary Figure 5. The results of 10 ChIP-Seq datasets for LDB1, RUNX1, ERG, FLI1, LMO2, and CEBPA in AML cell lines showed that those transcription factors bound to IRF2BP2 (track 1-10), and the Hi-C data analysis of THP-1 cell line (track 11) represents interaction at the IRF2BP2 gene loci.

Supplementary Figure 6. The results of 10 ChIP-Seq datasets for LDB1, RUNX1, ERG, FLI1, LMO2, and CEBPA in AML cell lines showed that those transcription factors bound to LYL1 (track 1-10), and the Hi-C data analysis of THP-1 cell line (track 11) represents interaction at the LYL1 gene loci.

Supplementary Table 1. Information about clinical patients involved in the experiment.

Supplementary Table 2. Results of LMO2 overexpression in 293T cells by mass spectrometry.

Supplementary Table 3. Deferentially expressed genes identified by RNA-Seq of NB4 cell after LDB1 knockdown.

Supplementary Table 4. HSC self-renewal associated genes down-regulated in NB4 cells knockdown LDB1.

Supplementary Table 5. Peaks called by ChIP-Seq of LDB1 in NB4 cell line.

Supplementary Table 6. 1101 genes which were identified both in ChIP-Seq and RNA-Seq.

Supplementary Table 7. 5052 genes which were associated with AML transcription factors in ≧ 8 ChIP-Seq datasets.

Supplementary Table 8. 200 genes which were commonly associated with super enhancers in ≧10 AML samples.

Supplementary Table 9. 19 candidate genes associated with AML transcription factors, super-enhancers and sensitive to LDB1 knockdown in AML cells.

Supplementary Table 10. The results of STR identification of the cell line involved in the experiment.
