# Supplementary figures and images for: LMO2 promotes the development of AML through interaction with transcription co-regulator LDB1
Source: Cell Death Dis. 2023 Aug 12;14(8):518. doi: 10.1038/s41419-023-06039-w (PMC10423285; doi:10.1038/s41419-023-06039-w)

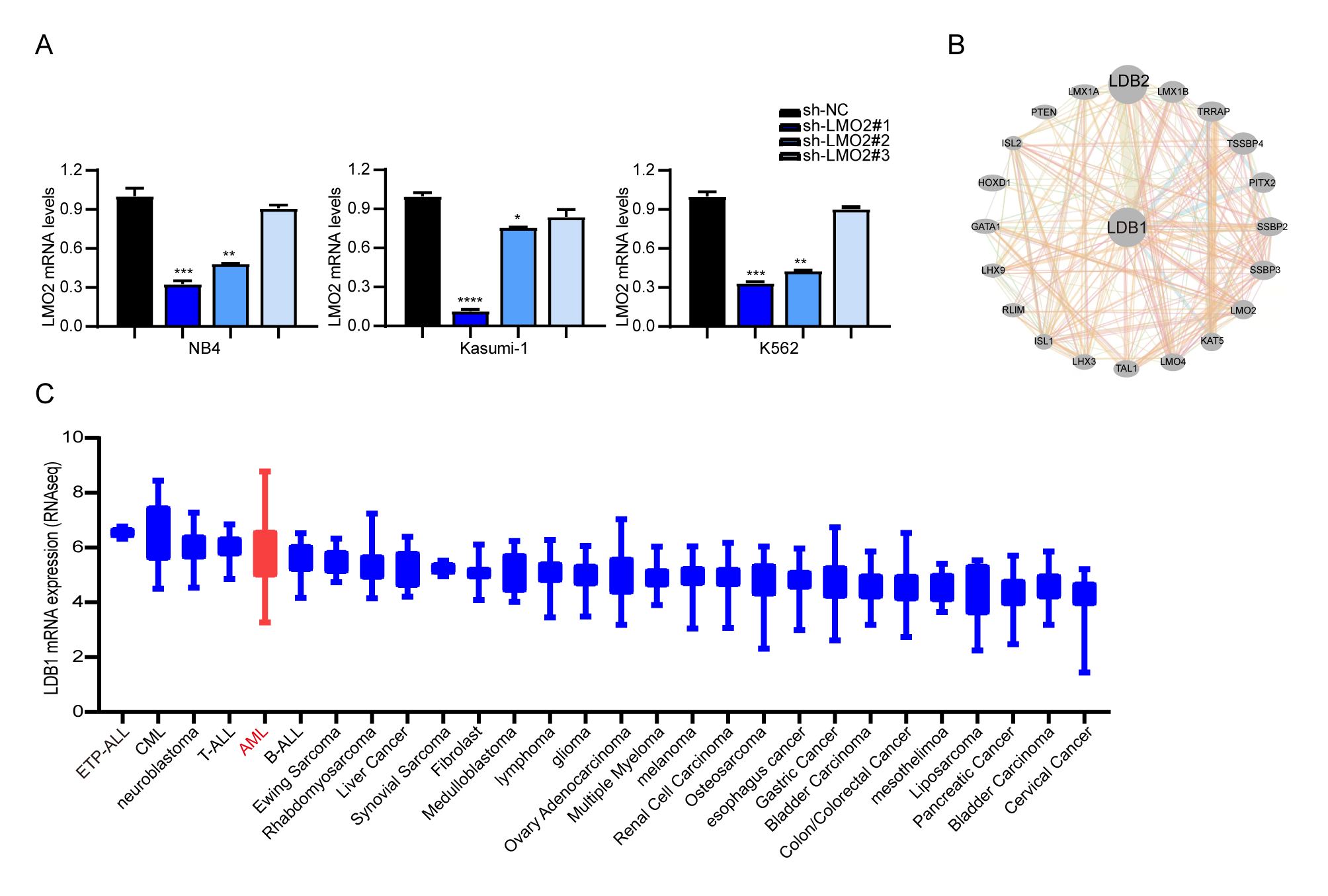

Supplement: Supplementary file 2 — Supplementary Figure 1 [file 41419_2023_6039_MOESM2_ESM.tif]

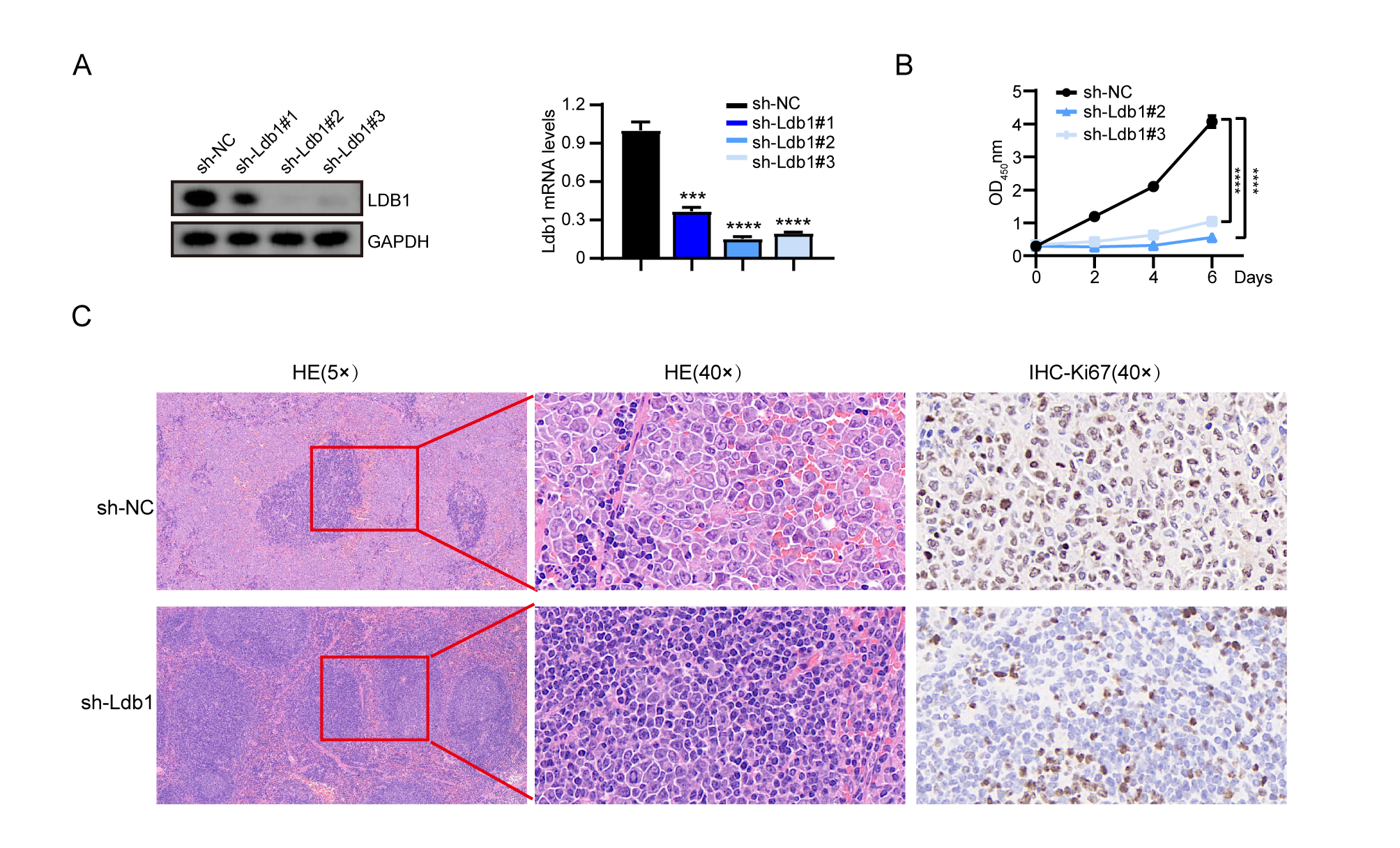

Supplement: Supplementary file 3 — Supplementary Figure 2 [file 41419_2023_6039_MOESM3_ESM.tif]

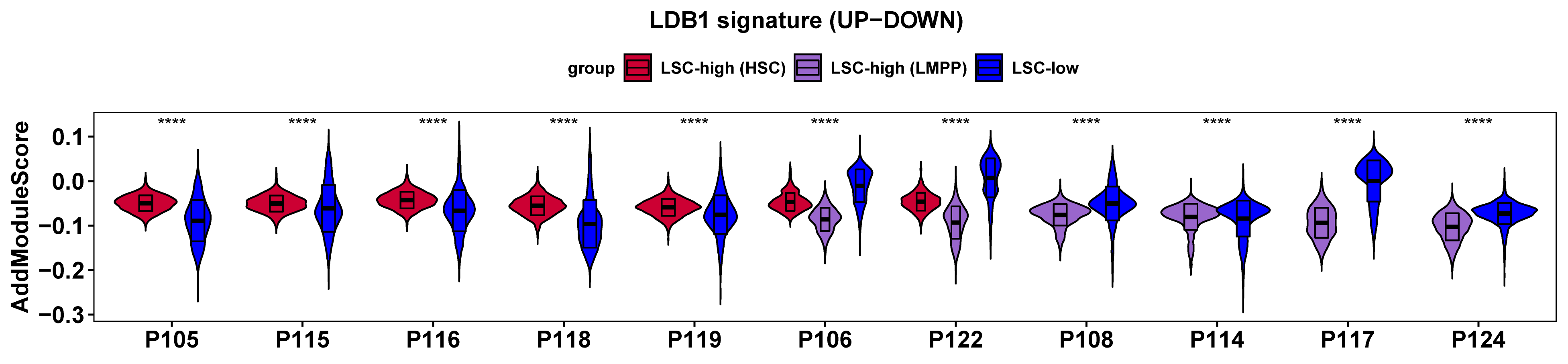

Supplement: Supplementary file 4 — Supplementary Figure 3 [file 41419_2023_6039_MOESM4_ESM.tif]

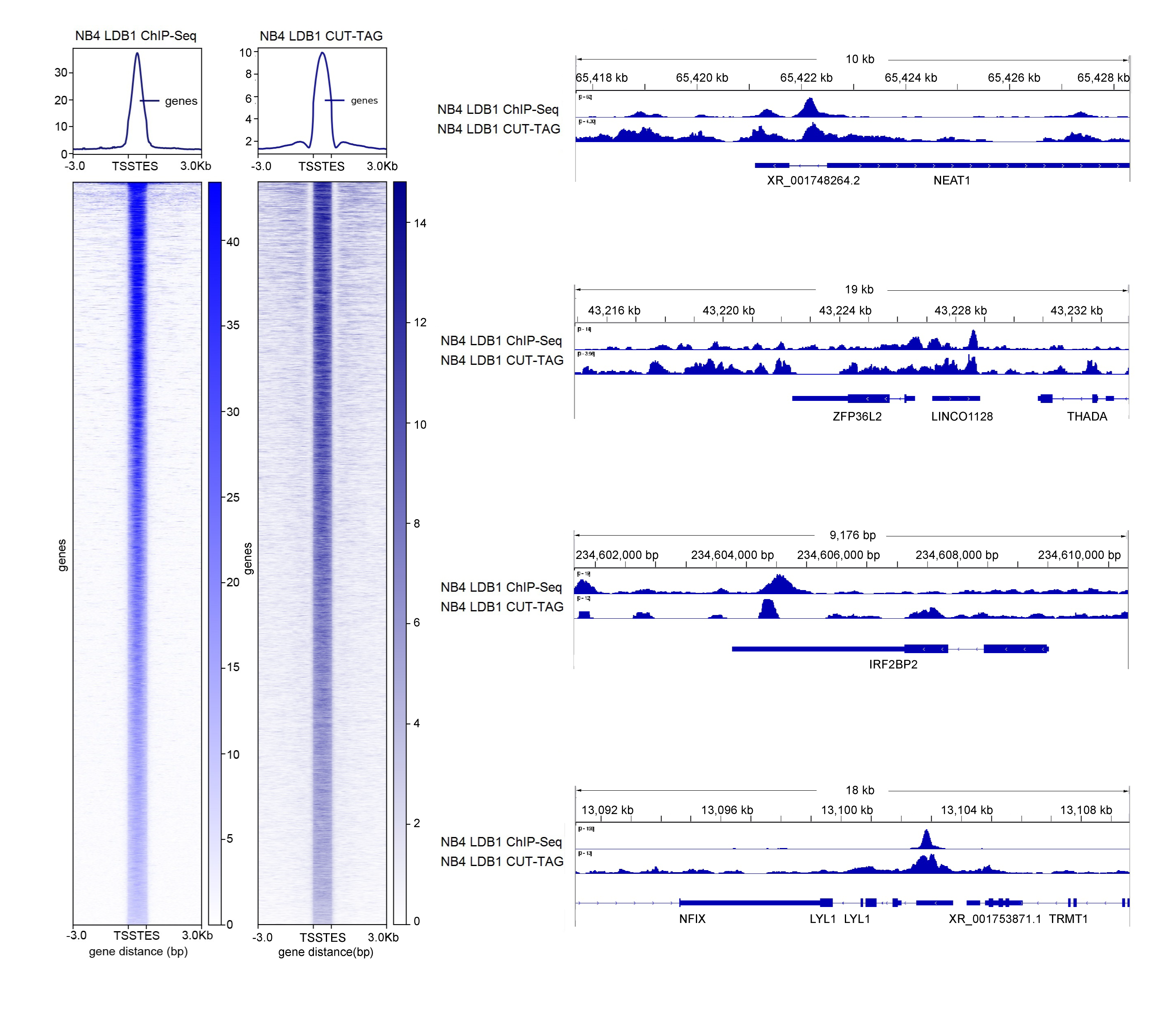

Supplement: Supplementary file 5 — Supplementary Figure 4 [file 41419_2023_6039_MOESM5_ESM.tif]

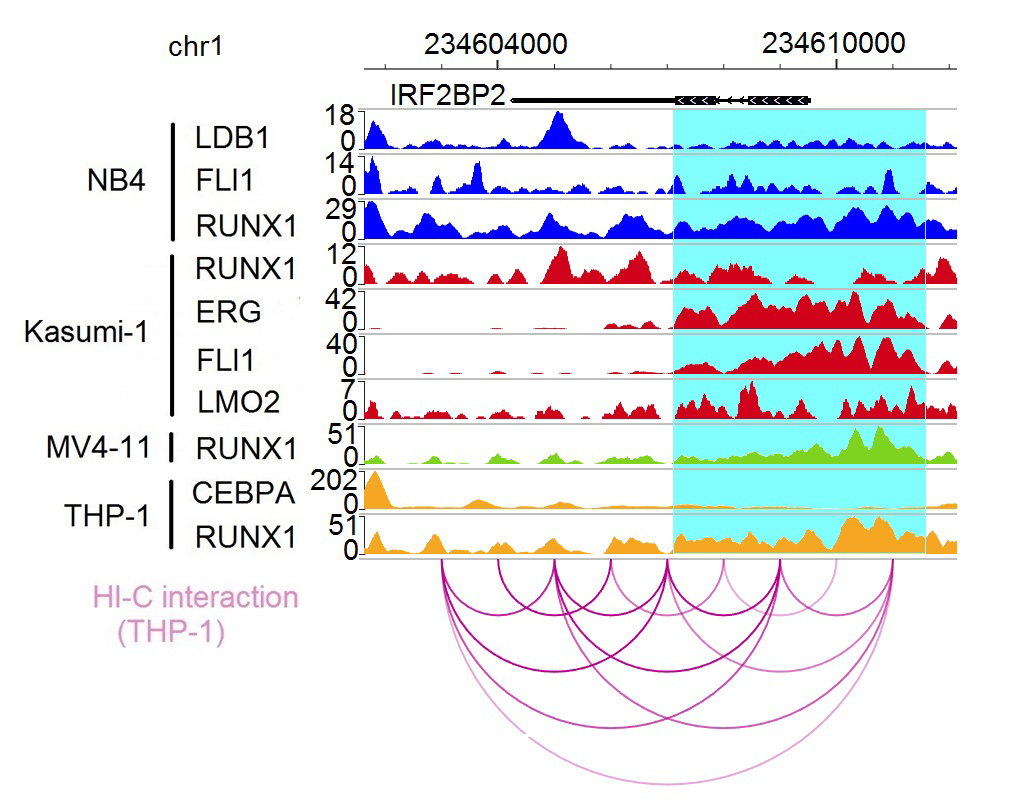

Supplement: Supplementary file 6 — Supplementary Figure 5 [file 41419_2023_6039_MOESM6_ESM.tif]

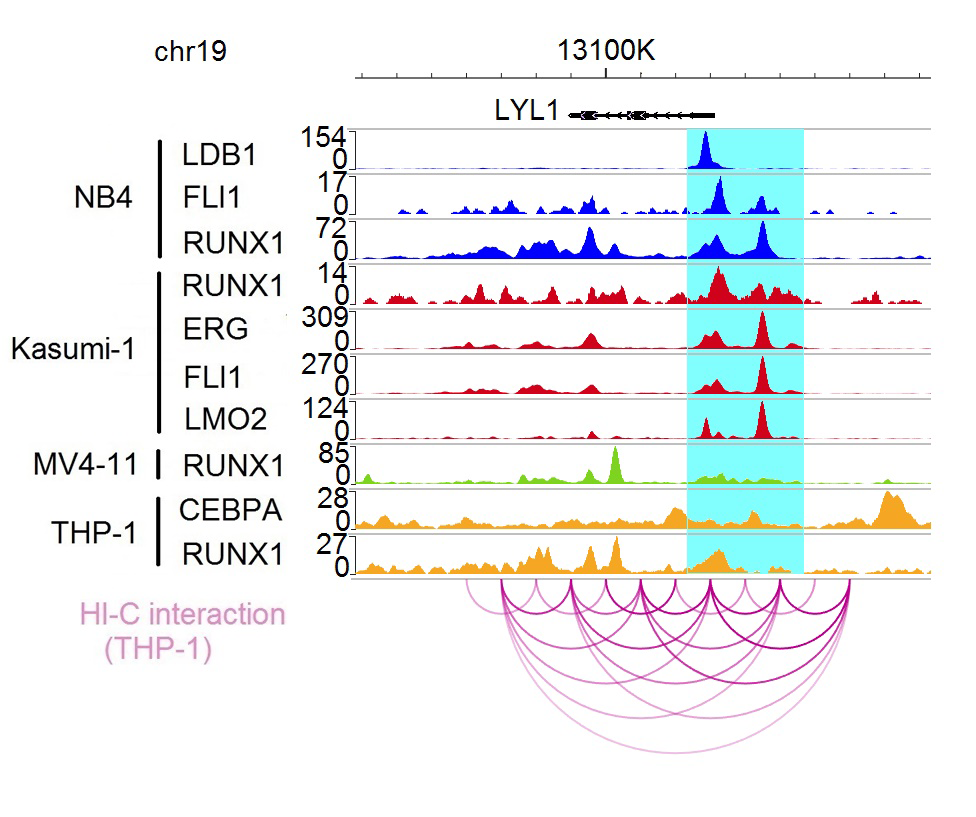

Supplement: Supplementary file 7 — Supplementary Figure 6 [file 41419_2023_6039_MOESM7_ESM.tif]
