# Supplementary material for: LMO2 promotes the development of AML through interaction with transcription co-regulator LDB1
Source: Cell Death Dis. 2023 Aug 12;14(8):518. doi: 10.1038/s41419-023-06039-w (PMC10423285; doi:10.1038/s41419-023-06039-w)
Supplement: Supplementary file 17 — Supplementary Table 10 [file 41419_2023_6039_MOESM17_ESM.pdf]

Cell Line Authentication Report

GENEWIZ, Inc.

C3 Building, 218 Xinghu Road

Suzhou Industrial Park, 215123

Suzhou, China

Tel: 400-8100-669

www.genewiz.com

苏州金唯智生物科技有限公司
中国苏州工业园区
星湖街218号纳米科技园C3楼 215123
电话:0512-68731001
传真:0512-62629530
www.genewiz.com.cn

Cell Line Authentication Report

Customer: Zuokang Zheng

Quotation Number: 80-446653207_R1

Completion Date: 05/14/2020

1. Sample ID: #24

2. Original Material: Cell Plate

3. Methods:

1).Genomic DNA was extracted from the cell pellets provided by the customer.

2).Samples, together with positive and negative control were amplified using GenePrint 10 System (Promega).

3). Amplified products were processed using the ABI3730xl Genetic Analyzer.

4). Data were analyzed using GeneMapper4.0 software and then compared with the ATCC, DSMZ, JCRB and RIKEN databases for reference matching.

4. Results:

1) 10 Loci STR Profile：

Genetic Site (Locus)	Customer sample #24	
Amelogenin	X	X
CSF1PO	9	10
D13S317	8	8
D16S539	11	12
D5S818	11	12
D7S820	9	11
THO1	9.3	9.3
TPOX	8	9
vWA	16	16
D21S11	29	30,31

苏州金唯智生物科技有限公司
中国苏州工业园区
星湖街218号纳米科技园C3楼 215123
电话:0512-68731001
传真:0512-62629530
www.genewiz.com.cn

<<< Search for reference matching with the Cell Bank databases and add the match results. >>>

Result of STR matching analysis by your data.
-DSMZ Profile Database-

EV	Cell No.	Cell name	Locus names								
			D5S818	D13S317	D7S820	D16S539	VWA	TH01	AM	TPOX	CSF1PO
	Query(Your Cell)		11,12	8,8	9,11	11,12	16,16	9.3,9.3	X,X	8,9	9,10
1.00(36/36)	CCL-243	K-562	11,12	8,8	9,11	11,12	16,16	9.3,9.3	X,X	8,9	9,10
1.00(36/36)	JCRB0019	K-562	11,12	8,8	9,11	11,12	16,16	9.3,9.3	X,X	8,9	9,10
1.00(36/36)	JCRB0122	KO51	11,12	8,8	9,11	11,12	16,16	9.3,9.3	X,X	8,9	9,10
1.00(36/36)	JCRB1002	K562/ADM	11,12	8,8	9,11	11,12	16,16	9.3,9.3	X,X	8,9	9,10
1.00(36/36)	RCB0027	K562	11,12	8,8	9,11	11,12	16,16	9.3,9.3	X,X	8,9	9,10
1.00(36/36)	RCB1197	P2UR/K-562	11,12	8,8	9,11	11,12	16,16	9.3,9.3	X,X	8,9	9,10
1.00(36/36)	RCB1898	K562/Adr	11,12	8,8	9,11	11,12	16,16	9.3,9.3	X,X	8,9	9,10
1.00(36/36)	RCB2111	K562/Vin	11,12	8,8	9,11	11,12	16,16	9.3,9.3	X,X	8,9	9,10
0.97(36/37)	10	K-562	11,12	8,8	9,11	11,12,13	16,16	9.3,9.3	X,X	8,9	9,10
0.95(36/38)	RCB1635	K-562	11,12,13	8,8	9,11	11,12,13	16,16	9.3,9.3	X,X	8,9	9,10
0.94(34/36)	RCB0474	K562/MTX-2	11,12	8,8	9,11	11,12	16,16	6,9.3	X,X	8,9	9,10
0.89(32/36)	86	SPI-801	12,12	8,8	9,11	11,12	16,16	9.3,9.3	X,X	8,9	10,10
0.89(32/36)	92	SPI-802	12,12	8,8	9,11	11,12	16,16	9.3,9.3	X,X	8,9	10,10
0.72(26/36)	677	BC-1	11,12	8,8	11,11	12,13	16,20	9.3,9.3	X,Y	8,9	10,11
0.72(26/36)	CRL-2230	BC-1	11,12	8,8	11,11	12,13	16,20	9.3,9.3	X,Y	8,9	10,11
0.72(26/36)	CRL-7443	Hs 704.Sk	11,12	11,12	8,10	11,12	16,16	9.3,9.3	X,X	8,9	10,12
0.72(26/36)	CRL-7444	Hs 704.T	11,12	11,12	8,10	11,12	16,16	9.3,9.3	X,X	8,9	10,12
0.67(24/36)	189	LN-405	11,12	8,8	9,11	10,10	15,16	8,9.3	X,X	8,11	10,11
0.67(24/36)	74	MEL-JUSO	11,12	8,9	10,12	12,13	16,16	9.3,9.3	X,X	8,10	10,11

Summary

Your cell line is considered “identical” to the reference cell line K-562 in the ATCC STR database, as the STR profile yields a 100% match.

GEN

2) Electrophoretogram

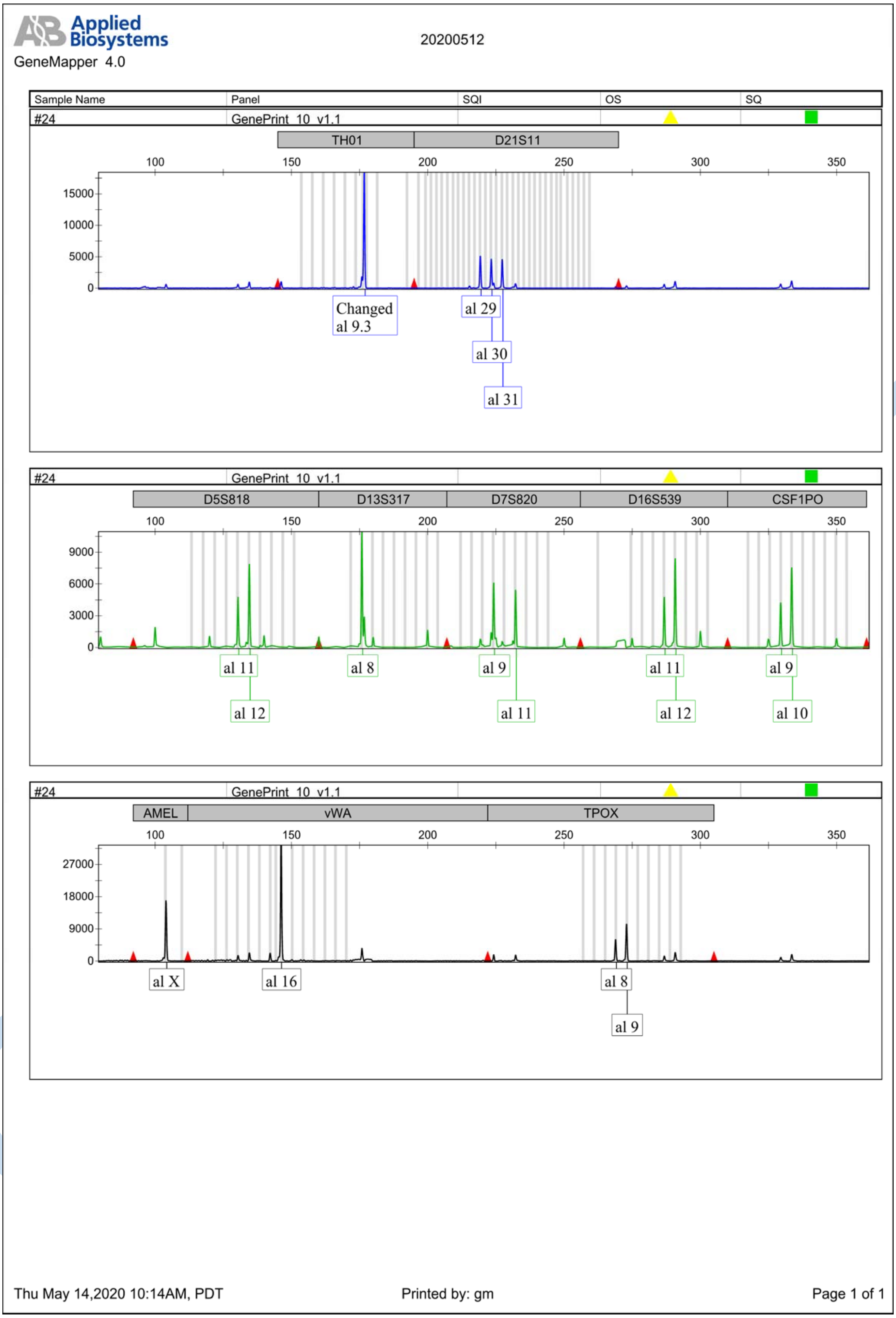

Note：Raw data in appendix

GENETIC TESTING BIOTECHNOLOGY

Cell Line Authentication Service
STR Profile Report

Sample Submitted By: Dr. Zuokang Zheng
Zhejiang Meisen Cell Technology Co.,Ltd.
Email Address: 947638289@qq.com
Sales Order: 210125A
Cell Line Designation: KASUMI-1
Date Sample Received: Jan 25th, 2021
Report Date: Jan 25th, 2021

Methodology: Nineteen short tandem repeat (STR) loci plus the gender determining locus, Amelogenin, were amplified using the commercially available EX20 Kit from AGCU. The cell line sample was processed using the ABI Prism® 3130 XL Genetic Analyzer. Data were analyzed using GeneMapper® ID v3.2 software (Applied Biosystems). Appropriate positive and negative controls were run and confirmed for each sample submitted.

Data Interpretation: Cell lines were authenticated using Short Tandem Repeat (STR) analysis as described in 2012 in ANSI Standard (ASN-0002) by the ATCC Standards Development Organization (SDO) and in Capes-Davis et al., Match criteria for human cell line authentication: Where do we draw the line? Int J Cancer. 2013;132(11):2510-9.

GTB™ performs STR Profiling following ISO 9001:2008 and ISO/IEC 17025:2005 quality standards.

There are no warranties with respect to the services or results supplied, express or implied, including, without limitation, any implied warranty of merchantability or fitness for a particular purpose. Genetic Testing Biotechnology (GTB) is not liable for any damages or injuries resulting from receipt and/or improper, inappropriate, negligent or other wrongful use of the test results supplied, and/or from misidentification, misrepresentation, or lack of accuracy of those results. Your exclusive remedy against GTB and those supplying materials used in the services for any losses or damage of any kind whatsoever, whether in contract, tort, or otherwise, shall be, at GTB's option, refund of the fee paid for such service or repeat of the service.

NOTE: According to the recommendations of *IJC* on cell line authentication, the report is valid for 3 years since the issue date.

Technical Questions?
GTB Technical Support
+86-512-67486171
service@jsdna.org
Section 505, Yixin BLD
SIP, Suzhou, 215123
Jiangsu, P.R. China

Ordering Questions?
order@jsdna.org
GTB Corporation
+86-512-62806339
Section 303, Yixin BLD
SIP, Suzhou, 215123
Jiangsu, P.R. China

GENETIC TESTING BIOTECHNOLOGY

Cell Line Authentication Service
STR Profile Report

Sales Order: 210125A

	Test Results for Submitted Sample		DSMZ Reference Database Profile	
**Loci**	**Query Profile:** KASUMI-1		**Database Profile:** KASUMI-1	
Amelogenin	X		X	
D3S1358	15	17		
D13S317	11	13	11	13
D7S820	8	11	8	11
D16S539	9	12	9	12
Penta E	11			
TPOX	8	9	8	9
TH01	6	9	6	9
D2S1338	19			
CSF1PO	10	12	10	12
Penta D	12			
D19S433	13	15.2		
vWA	14		14	
D21S11	30	31		
D18S51	15	16		
D6S1043	19	20.3		
D8S1179	13	14		
D5S818	9	11	9	11
D12S391	20			
FGA	22	24		

The allele match algorithm compares the 8 core loci plus amelogenin only, even though alleles from all loci will be reported when available.

*Note: Loci highlighted in grey (8 core STR loci plus Amelogenin) can be made public to verify cell identity. In order to protect the identity of the donor, **please do not publish** the allele calls from all the STR loci tested. The sample match is based on the reference data available at the time of comparison.*

Explanation of Test Results

Cell lines with ≥80% match are considered to be related; i.e., derived from a common ancestry. Cell lines with between a 55% to 80% match require further profiling for authentication of relatedness.

☐ The submitted sample profile is human, but not a match for any profile in the DSMZ STR database.

☑ The submitted profile is an exact match for the following human cell line(s) in the DSMZ STR database (8 core loci plus Amelogenin): KASUMI-1

☐ The submitted profile is similar to the following DSMZ human cell line(s):

e-Signature, Technician:

e-Signature, Reviewer:

Addendum: Electropherogram for the customer's sample set 1 of 1

Cell Line Authentication Service
STR Profile Report

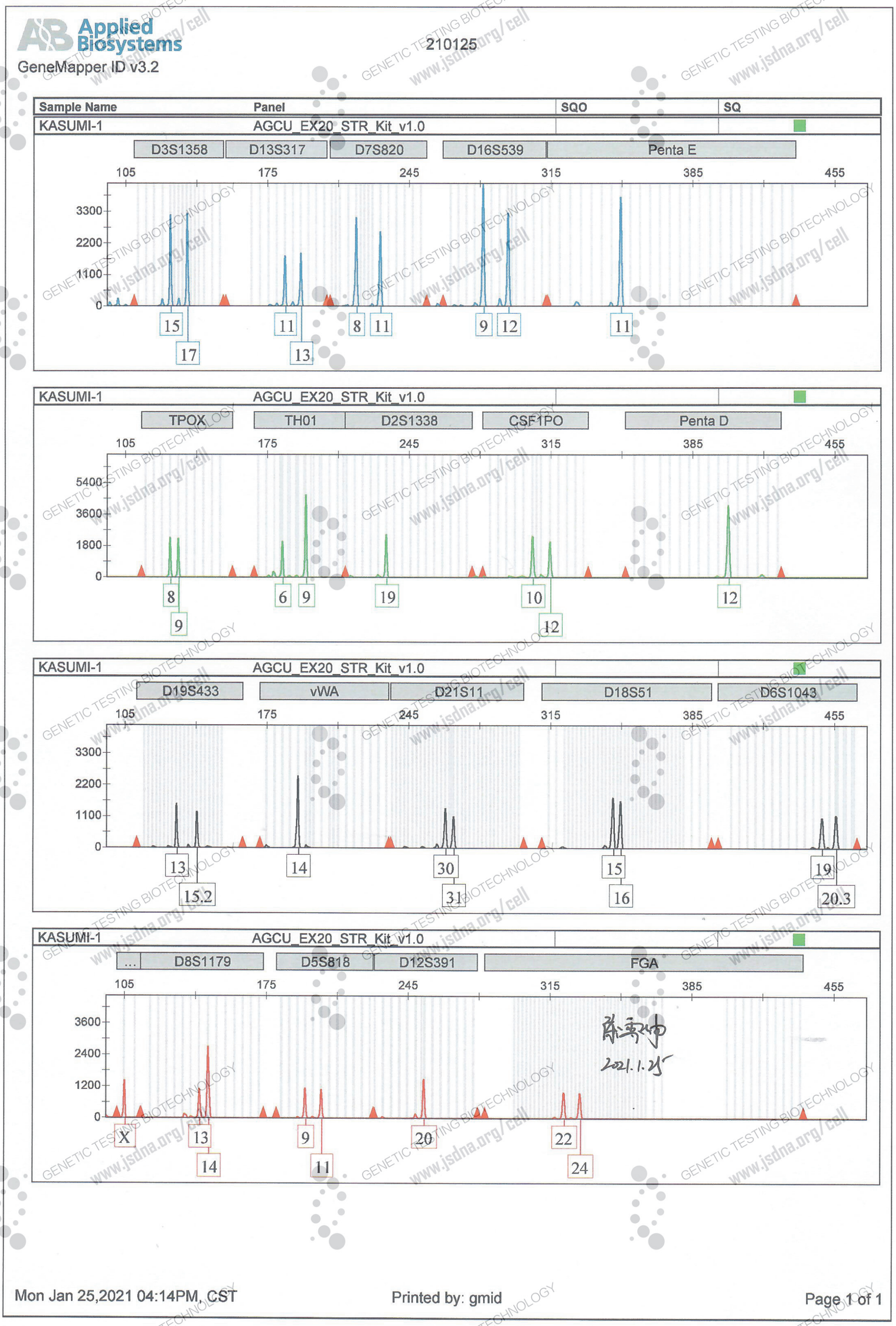

GENETIC TESTING BIOTECHNOLOGY

Cell Line Authentication Service
STR Profile Report

Result of STR matching analysis by your data.

- DSMZ Profile Database -

A graphical presentation is shown at the bottom of this page.

EV	Cell No.	Cell name	Locus names									Figures
			D5S818	D13S317	D7S820	D16S539	VWA	TH01	AM	TPOX	CSF1PO	
	Query (Your Cell)		9,11	11,13	8,11	9,12	14,14	6,9	x,x	8,9	10,12	
1.00(36/36)	220	KASUMI-1	9,11	11,13	8,11	9,12	14,14	6,9	X,X	8,9	10,12	-
1.00(36/36)	CRL-2724	Kasumi-1	9,11	11,13	8,11	9,12	14,14	6,9	X,X	8,9	10,12	-
1.00(36/36)	JCRB1003	Kasumi-1	9,11	11,13	8,11	9,12	14,14	6,9	X,X	8,9	10,12	-
0.72(26/36)	RCB0587	SF8759	11,12	11,12	10,11	9,9	14,18	6,9	X,X	8,9	10,12	-
0.67(24/36)	535	CL-40	11,11	8,11	8,9	11,12	16,17	6,9	X,X	8,9	10,12	-
0.67(24/36)	CRL-7473	Hs 737.T	9,11	11,11	8,12	9,11	16,18	6,9	X,X	8,9	10,10	-
0.67(24/36)	IFO50436	KS-1	8,12	11,13	11,11	11,12	14,18	6,9	X,X	8,11	10,12	-
0.67(24/36)	JCRB0834	NUGC-4	11,12	11,13	11,11	9,9	14,18	7,9	X,X	8,9	10,11	-
0.67(24/36)	JCRB1114	Yub622	11,13	11,13	11,11	9,12	14,18	6,9.3	X,X	8,8	10,11	-
0.67(24/36)	RCB0520	WR216	10,13	12,14	8,12	9,12	14,14	6,9	X,X	8,9	10,11	-
0.67(24/36)	RCB1939	NUGC-4	11,12	11,13	11,11	9,9	14,18	7,9	X,X	8,9	10,11	-
0.67(24/36)	RCB2352	633	11,11	11,11	8,11	11,12	14,14	8,9.3	X,X	8,11	10,12	-
0.65(24/37)	JCRB1030	JHH-6	10,11	11,12,13	11,11	10,10	14,18	7,9	X,X	8,9	10,12	-
0.61(22/36)	107	A-549	11,11	11,11	8,11	11,12	14,14	8,9.3	X,Y	8,11	10,12	-
0.61(22/36)	255	CADO-ES1	11,12	10,13	11,13	9,11	14,18	6,9	X,X	8,11	11,12	-
0.61(22/36)	294	SW-403	11,11	13,13	8,9	10,12	14,18	6,6	X,X	8,9	10,13	-
0.61(22/36)	430	SK-MM-2	9,11	10,10	8,11	11,11	14,20	6,6	X,X	8,11	10,12	-
0.61(22/36)	444	FU-OV-1	11,13	8,10	11,11	12,12	14,19	6,6	X,X	8,9	10,12	-
0.61(22/36)	552	KOPN-8	9,11	8,11	8,10	9,11	16,18	6,10	X,X	8,11	10,12	-
0.61(22/36)	573	SU-DHL-8	11,13	11,13	8,8	12,12	15,19	6,9	X,X	8,8	11,12	-
0.61(22/36)	580	HTC-C3	11,12	10,12	11,12	9,12	14,15	6,7	X,X	8,9	11,12	-
0.61(22/36)	600	HNT-34	13,13	10,11	8,11	9,12	14,14	6,9	X,X	11,11	11,13	-
0.61(22/36)	723	TK-6	11,12	8,13	8,11	9,12	15,16	6,9	X,Y	8,8	10,11	-
0.61(22/36)	CCL-185	A549	11,11	11,11	8,11	11,12	14,14	8,9.3	X,Y	8,11	10,12	-
0.61(22/36)	CCL-230	SW403 [SW-403]	11,11	13,13	8,9	10,12	14,18	6,6	X,X	8,9	10,13	-
0.61(22/36)	CRL-2089	CCD-1069Sk	9,11	8,11	9,10	12,13	16,18	6,9	X,X	8,8	10,12	-
0.61(22/36)	CRL-2719	11	9,12	12,12	11,11	9,14	16,17	6,9	X,X	8,9	10,12	-

Cell Line Authentication Service
STR Profile Report

Sample Submitted By: Dr. Zuokang Zheng
Zhejiang Meisen Cell Technology Co.,Ltd.
Email Address: 947638289@qq.com
Sales Order: 210408W
Cell Line Designation: NB-4
Date Sample Received: Apr 8th, 2021
Report Date: Apr 9th, 2021

Methodology: Nineteen short tandem repeat (STR) loci plus the gender determining locus, Amelogenin, were amplified using the commercially available EX20 Kit from AGCU. The cell line sample was processed using the ABI Prism® 3130 XL Genetic Analyzer. Data were analyzed using GeneMapper® ID v3.2 software (Applied Biosystems). Appropriate positive and negative controls were run and confirmed for each sample submitted.

Data Interpretation: Cell lines were authenticated using Short Tandem Repeat (STR) analysis as described in 2012 in ANSI Standard (ASN-0002) by the ATCC Standards Development Organization (SDO) and in Capes-Davis et al., Match criteria for human cell line authentication: Where do we draw the line? Int J Cancer. 2013;132(11):2510-9.

GTB™ performs STR Profiling following ISO 9001:2008 and ISO/IEC 17025:2005 quality standards.
There are no warranties with respect to the services or results supplied, express or implied, including, without limitation, any implied warranty of merchantability or fitness for a particular purpose. Genetic Testing Biotechnology (GTB) is not liable for any damages or injuries resulting from receipt and/or improper, inappropriate, negligent or other wrongful use of the test results supplied, and/or from misidentification, misrepresentation, or lack of accuracy of those results. Your exclusive remedy against GTB and those supplying materials used in the services for any losses or damage of any kind whatsoever, whether in contract, tort, or otherwise, shall be, at GTB's option, refund of the fee paid for such service or repeat of the service.

NOTE: According to the recommendations of *IJC* on cell line authentication, the report is valid for 3 years since the issue date.

Technical Questions?
GTB Technical Support
+86-512-67486171
service@jsdna.org
Section 505, Yixin BLD
SIP, Suzhou, 215123
Jiangsu, P.R. China

Ordering Questions?
order@jsdna.org
GTB Corporation
+86-512-62806339
Section 303, Yixin BLD
SIP, Suzhou, 215123
Jiangsu, P.R. China

GENETIC TESTING BIOTECHNOLOGY

Cell Line Authentication Service
STR Profile Report

Sales Order: 210408W

	Test Results for Submitted Sample		DSMZ Reference Database Profile	
**Loci**	**Query Profile:** NB-4		**Database Profile:** NB-4	
Amelogenin	X		X	
D3S1358	15	17		
D13S317	11	12	11	12
D7S820	10	13	10	13
D16S539	9		9	
Penta E	7	13		
TPOX	8	11	8	11
TH01	7	9.3	7	9.3
D2S1338	20	25		
CSF1PO	11	12	11	12
Penta D	10	13		
D19S433	15			
vWA	16	19	16	19
D21S11	28	33.2		
D18S51	12			
D6S1043	12			
D8S1179	10	14		
D5S818	13		13	
D12S391	19	22		
FGA	21	22		

The allele match algorithm compares the 8 core loci plus amelogenin only, even though alleles from all loci will be reported when available.

Note: Loci highlighted in grey (8 core STR loci plus Amelogenin) can be made public to verify cell identity. In order to protect the identity of the donor, ***please do not publish*** *the allele calls from all the STR loci tested. The sample match is based on the reference data available at the time of comparison.*

Explanation of Test Results

Cell lines with ≥80% match are considered to be related; i.e., derived from a common ancestry. Cell lines with between a 55% to 80% match require further profiling for authentication of relatedness.

☐ The submitted sample profile is human, but not a match for any profile in the DSMZ STR database.

☑ The submitted profile is an exact match for the following human cell line(s) in the DSMZ STR database (8 core loci plus Amelogenin): NB-4

☐ The submitted profile is similar to the following DSMZ human cell line(s):

e-Signature, Technician:

e-Signature, Reviewer:

Addendum: Electropherogram for the customer's sample set 1 of 1

Cell Line Authentication Service
STR Profile Report

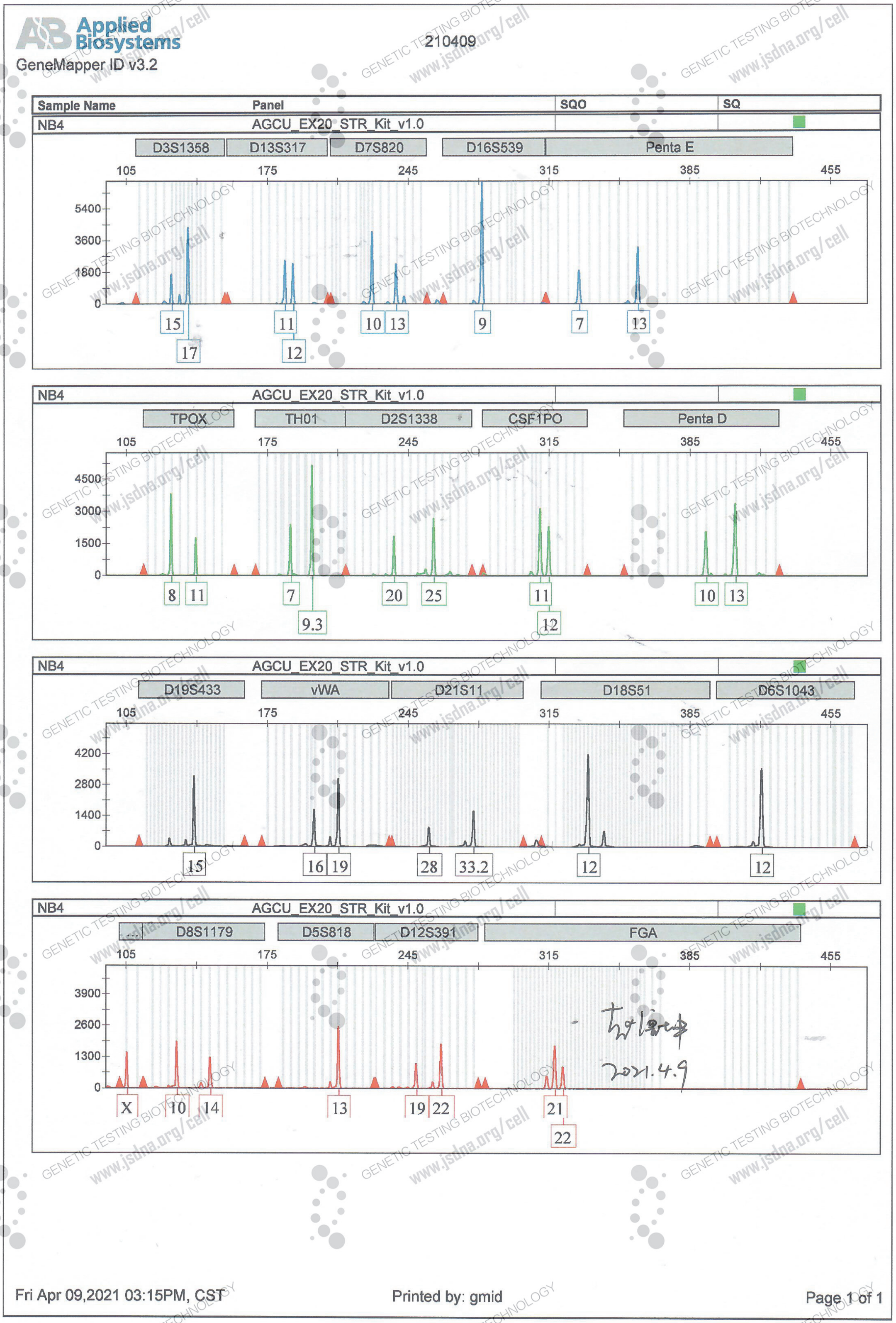

Result of STR matching analysis by your data.

- DSMZ Profile Database -

A graphical presentation is shown at the bottom of this page.

EV	Cell No.	Cell name	D5S818	D13S317	D7S820	D16S539	VWA	TH01	AM	TPOX	CSF1PO	Figures
	Query (Your Cell)		13,13	11,12	10,13	9,9	16,19	7,9.3	X,X	8,11	11,12	
1.00(36/36)	207	NB-4	13,13	11,12	10,13	9,9	16,19	7,9.3	X,X	8,11	11,12	-
0.72(26/36)	CRL-2056	CCD-1043Sk	13,13	11,12	10,10	9,11	16,19	7,7	X,Y	8,11	12,12	-
0.72(26/36)	JCRB1092	NCE SVIA6	10,13	11,11	10,13	9,9	16,17	7,7	X,X	8,11	10,12	-
0.72(26/36)	RCB1706	JHCOLOYI	12,13	11,12	10,12	9,9	16,17	7,9	X,X	8,11	9,11	-
0.67(24/36)	71	NAMALWA.IPN/45	12,13	11,12	11,11	9,9	14,16	7,9.3	X,X	6,11	10,11	-
0.67(24/36)	278	DLD-1	13,13	8,11	10,12	12,13	18,19	7,9.3	X,Y	8,11	11,12	-
0.67(24/36)	305	293	8,9	12,14	11,12	9,9	16,19	7,9.3	X,X	11,11	11,12	-
0.67(24/36)	433	EVSA-T	12,13	11,12	10,12	9,9	15,16	6,6	X,X	8,8	11,12	-
0.67(24/36)	436	MOLT-13	12,12	11,12	10,13	9,11	17,19	8,9.3	X,X	8,8	11,12	-
0.67(24/36)	655	LAN-1	12,12	11,12	10,11	9,9	16,19	8,9.3	X,Y	8,11	12,12	-
0.67(24/36)	CCL-221	DLD-1	13,13	8,11	10,12	12,13	18,19	7,9.3	X,Y	8,11	11,12	-
0.67(24/36)	CRL-10852	293 c18	8,9	12,14	11,12	9,9	16,19	7,9.3	X,X	11,11	11,12	-
0.67(24/36)	CRL-11654	90,74	8,9	12,14	11,12	9,9	16,19	7,9.3	X,X	11,11	11,12	-
0.67(24/36)	CRL-12007	ProPak-X.36 [PP-X.36]	8,9	12,14	11,12	9,9	16,19	7,9.3	X,X	11,11	11,12	-
0.67(24/36)	CRL-12386	SODK1	8,9	12,14	11,12	9,9	16,19	7,9.3	X,X	11,11	11,12	-
0.67(24/36)	CRL-12479	ProPak-A.52 Clone #52 [PP-A.52]	8,9	12,14	11,12	9,9	16,19	7,9.3	X,X	11,11	11,12	-
0.67(24/36)	CRL-1881	CCD-966Sk	12,13	11,12	10,10	9,12	16,19	7,8	X,X	9,11	10,12	-
0.67(24/36)	CRL-2319	HCC 1007BL	13,13	11,12	10,10	9,14	17,19	7,8	X,X	6,11	12,13	-
0.67(24/36)	CRL-2828	PEAKrapid	8,9	12,14	11,12	9,9	16,19	7,9.3	X,X	11,11	11,12	-
0.67(24/36)	CRL-3022	HEK 293S GnTI-	8,9	12,14	11,12	9,9	16,19	7,9.3	X,X	11,11	11,12	-
0.67(24/36)	CRL-5837	NCI-H719 [H719]	13,13	12,12	10,10	9,9	16,18	8,9	X,X	8,11	10,11	-
0.67(24/36)	JCRB9094	DLD-1	13,13	8,11	10,12	12,13	18,19	7,9.3	X,Y	8,11	11,12	-
0.67(24/36)	RCB0483	LA-N-1	12,12	11,12	10,11	9,9	16,19	8,9.3	X,Y	8,11	12,12	-
0.67(24/36)	RCB1384	HCE-T	13,13	8,8	10,13	9,11	18,19	7,8	X,X	8,11	12,12	-

细胞遗传质量鉴定检测

Cell Line Authentication Service

STR 基因型检测报告

送检单位： 苏州纳金肽

检品名称： 细胞系

委托单位： 上海翼和应用生物技术有限公司

报告日期： 2023/01/09

上海翼和应用生物技术有限公司联系地址：上海市松江区龙腾路 1015 弄中星创意园 2 号 502 室
网址：http://www.biowing.com.cn/邮箱：market@biowing.com.cn 服务热线：18521538068

报 告 说 明

1. 本报告只对送检的来样负责。
2. 检验报告上的检验结果和检验单位名称，未经同意不得用于广告、评优及商业宣传。
3. 对本报告有异议，请于收到报告之日起十五日内以书面方式提出，逾期不予受理。
4. 对纸质检验报告涂改、增删，或未加盖检验单位印章的复印件均无效。

上海翼和应用生物技术有限公司联系地址：上海市松江区龙腾路 1015 弄中星创意园 2 号 502 室
网址：http://www.biowing.com.cn/邮箱：market@biowing.com.cn 服务热线：18521538068

样 品 信 息

样品编号:

客户样本编号	公司编号
P388D1	20220104-01

样品数量: 1

样品性状: 细胞系

检测项目: STR

送检单位: 苏州纳金肽

检测方法: 用 Axygen 的基因组抽提试剂盒提取 DNA，采用 10- STR 扩增方案扩增，在 ABI 3730XL 型遗传分析仪上对 STR 位点和性别基因 Amelogenin 进行检测。

上海翼和应用生物技术有限公司联系地址：上海市松江区龙腾路 1015 弄中星创意园 2 号 502 室
网址：http://www.biowing.com.cn/邮箱：market@biowing.com.cn 服务热线：18521538068

检 测 结 果

(一) 检验基本情况

编号	多等位基因	匹配细胞系	人源污染	与对比细胞匹配度 EV 值	匹配说明
20220104-01	无	鼠源 P388D1	无	1.0	完全匹配

样本基因型检验结果

- 多等位基因指三等位及以上基因现象。
- 本次检测各细胞分型结果良好。

(二) 各样本描述

- 20220104-01：该株细胞鉴定结果为小鼠细胞系，细胞 STR 分型结果与对照细胞系 P388D1 基因型一致，细胞号对应 CVCL_0477，STR 分型结果完全匹配。本次检测在该细胞系中未发现多等位基因，无交叉污染，无人源污染。
- **备注：**待测细胞系与收录于 ATCC, DSMZ, J CRB 和 RIKEN 数据库的细胞系 STR 数据进行比对，未收录于以上细胞库的细胞系将无法匹配。下列位点中 D4S2408 为人源位点，用于检测该细胞是否有人源污染。

（三）样本分型结果

细胞 20220104-01 的 STR 位点和 Amelogenin 位点的基因分型结果

Loci	送检细胞 STR 信息				细胞库细胞 STR 信息		
	送检细胞名：P388D1				细胞库细胞名：P388D1		
	Allele1	Allele2	Allele3	Allele4	Allele1	Allele2	Allele3
4-2	238.19【20.3】				237.35【20.3】		
5-5	332.03【13】	336.05【14】			331.28【13】	335.3【14】	
6-4	296.37【17】				295.69【17】		
6-7	334.63【12】				333.95【12】		
9-2	221.71【15】				220.9【15】		
12-1	226.38【16】				225.27【16】		
15-3	197.27【21.3】	201.39【22.3】			196.53【21.3】	200.52【22.3】	
18-3	156.53【17】	160.61【18】			155.61【17】	159.57【18】	
X-1	408.84【27】				408.1【27】		
D4S2408							

上海翼和应用生物技术有限公司联系地址：上海市松江区龙腾路 1015 弄中星创意园 2 号 502 室
网址：http://www.biowing.com.cn/邮箱：market@biowing.com.cn 服务热线：18521538068

其 他 说 明

(一) 分型方案及位点分布

	方案 1	方案 2
1	18-3(FAM)	12-1(FAM)
2	4-2 (FAM)	5-5(FAM)
3	6-7(FAM)	X-1(FAM)
4	9-2(NED)	15-3(NED)
5		6-4(NED)
6		D4S2408(NED)

实验方案及位点

主要实验人员：张佳男

复核人: 张晨茜

负责人： 白杨

签发日期：2023 年 01 月 09 日

上海翼和应用生物技术有限公司联系地址：上海市松江区龙腾路 1015 弄中星创意园 2 号 502 室
网址：http://www.biowing.com.cn/邮箱：market@biowing.com.cn 服务热线：18521538068
