# Supplementary material for: LMO2 promotes the development of AML through interaction with transcription co-regulator LDB1
Source: Cell Death Dis. 2023 Aug 12;14(8):518. doi: 10.1038/s41419-023-06039-w (PMC10423285; doi:10.1038/s41419-023-06039-w)

Figure 1D-GAPDH

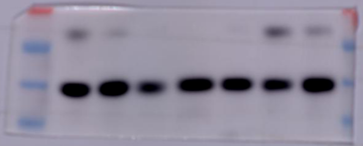

Figure 1D-LMO2

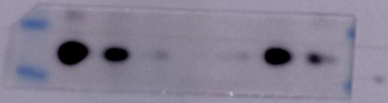

Figure 2A-K562-GAPDH

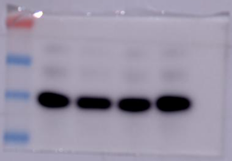

Figure 2A-K562-LMO2

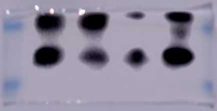

Figure 2A-KASUMI-GAPDH

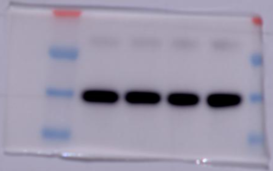

Figure 2A-KASUMI-LMO2

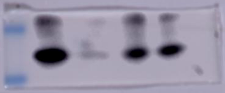

Figure 2A-NB4-GAPDH

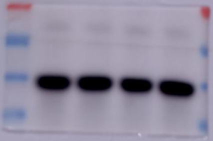

Figure 2A-NB4-LMO2

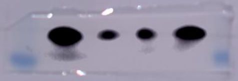

Figure 2C-K562-CMYC

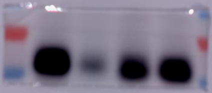

Figure 2C-K562-GAPDH

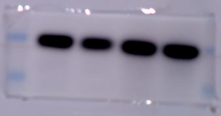

Figure 2C-K562-PARP

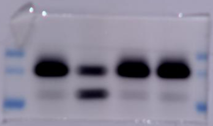

Figure 2C-KASUMI-CMYC

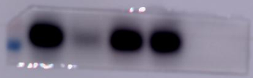

Figure 2C-KASUMI-GAPDH

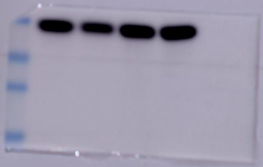

Figure 2C-KUSUMI-PARP

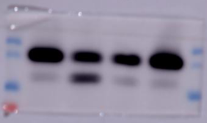

Figure 2C-NB4-CMYC

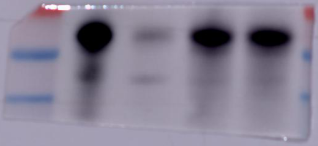

Figure 2C-NB4-GAPDH

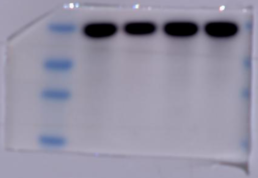

Figure 2C-NB4-PARP

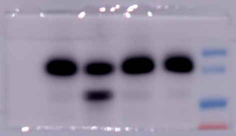

Figure 3C-IP-HA-GAPDH

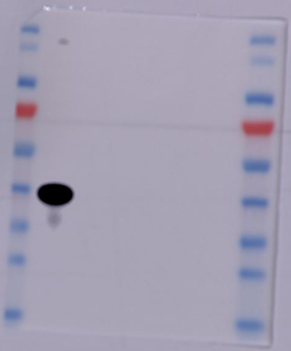

Figure 3C-IP-HA-LDB1

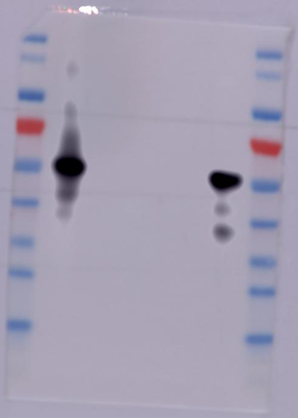

Figure 3C-IP-HA-LMO2

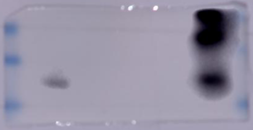

Figure 3C-IP-LDB1-GAPDH

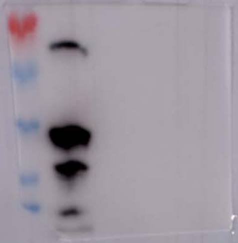

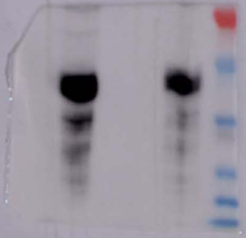

Figure 3C-IP-LDB1-LDB1

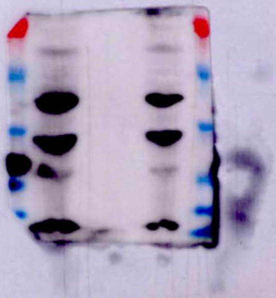

Figure 3C-IP-LDB1-LMO2

Figure 3D-GAPDH

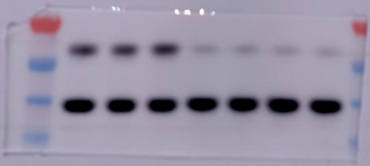

Figure 3D-LDB1

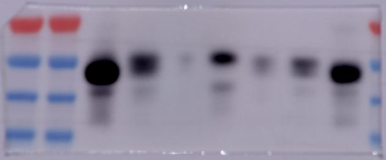

Figure 3E-K562-GAPDH

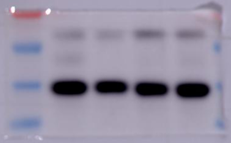

Figure 3E-K562-LDB1

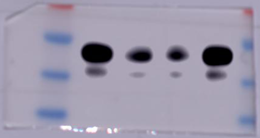

Figure 3E-KUSUMI-GAPDH

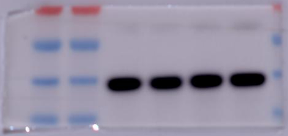

Figure 3E-KUSUMI-LDB1

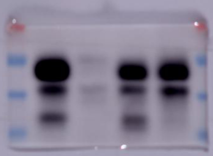

Figure 3E-NB4-GAPDH

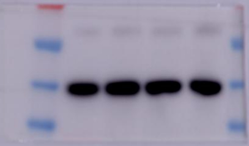

Figure 3E-NB4-LDB1

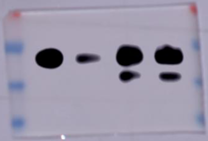

Figure 4D-K562-CMYC

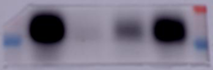

Figure 4D-K562-GAPDH

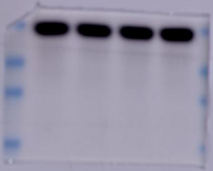

Figure 4D-K562-PARP

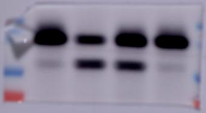

Figure 4D-KUSUMI-CMYC

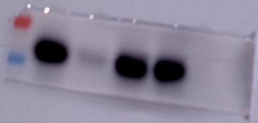

Figure 4D-KUSUMI-GAPDH

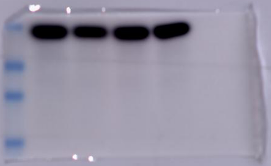

Figure 4D-KUSUMI-PARP

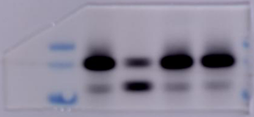

Figure 4D-NB4-CMYC

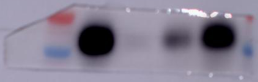

Figure 4D-NB4-GAPDH

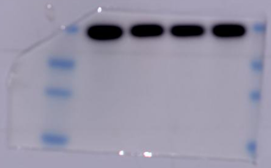

Figure 4D-NB4-PARP

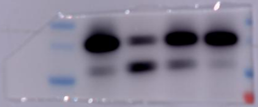

Figure 8A-sample1-sample2-GAPDH

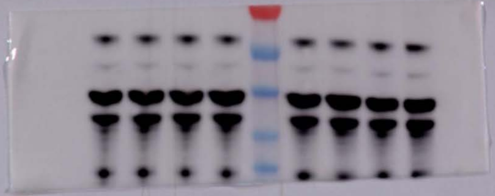

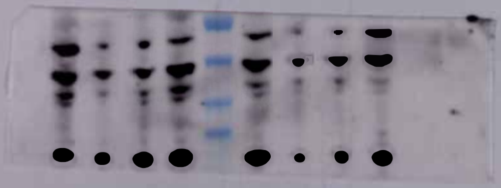

Figure 8A-sample1-sample2-LMO2

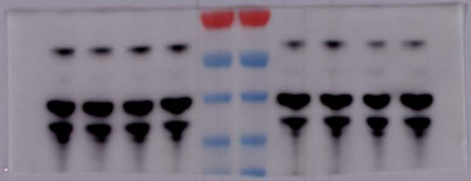

Figure 8B-sample1-sample2-GAPDH

Figure 8B-sample1-sample2-LDB1

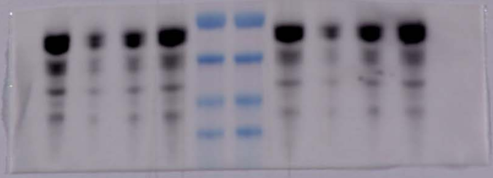

Figure 8C-CMYC

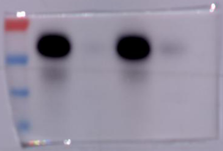

Figure 8C-GAPDH

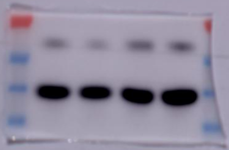

Figure 8C-LDB1

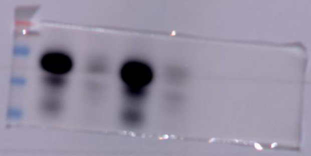

Figure 8C-LMO2

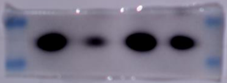

Figure 8C-NB4-PARP

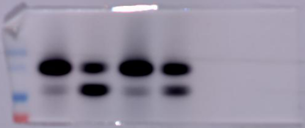

supplementary figure 2A-GAPDH

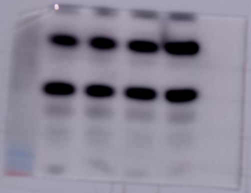

supplementary figure 2A-LDB1

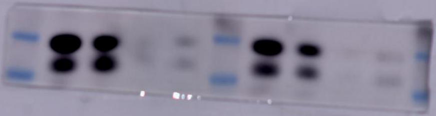

Supplement: Supplementary file 18 — Original Data File [file 41419_2023_6039_MOESM18_ESM.pdf]
